# The Putative Endonuclease Activity of MutL Is Required for the Segmental Gene Conversion Events That Drive Antigenic Variation of the Lyme Disease Spirochete

**DOI:** 10.3389/fmicb.2022.888494

**Published:** 2022-05-19

**Authors:** Mildred Castellanos, Theodore B. Verhey, Madeleine Goldstein, George Chaconas

**Affiliations:** ^1^Department of Biochemistry and Molecular Biology, Cumming School of Medicine, Snyder Institute for Chronic Diseases, University of Calgary, Calgary, AB, Canada; ^2^Department of Microbiology, Immunology and Infectious Diseases, Cumming School of Medicine, Snyder Institute for Chronic Diseases, University of Calgary, Calgary, AB, Canada

**Keywords:** Lyme disease, borreliosis, *Borrelia*, antigenic variation, *vlsE*, gene conversion, *mutL*

## Abstract

The Lyme disease spirochete *Borrelia burgdorferi*, encodes an elaborate antigenic variation system that promotes the ongoing variation of a major surface lipoprotein, VlsE. Changes in VlsE are continual and always one step ahead of the host acquired immune system, which requires 1–2 weeks to generate specific antibodies. By the time this happens, new VlsE variants have arisen that escape immunosurveillance, providing an avenue for persistent infection. This antigenic variation system is driven by segmental gene conversion events that transfer information from a series of silent cassettes (*vls2-16)* to the expression locus, *vlsE.* The molecular details of this process remain elusive. Recombinational switching at *vlsE* is RecA-independent and the only required factor identified to date is the RuvAB branch migrase. In this work we have used next generation long-read sequencing to analyze the effect of several DNA replication/recombination/repair gene disruptions on the frequency of gene conversions at *vlsE* and report a requirement for the mismatch repair protein MutL. Site directed mutagenesis of *mutL* suggests that the putative MutL endonuclease activity is required for recombinational switching at *vlsE.* This is the first report of an unexpected essential role for MutL in a bacterial recombination system and expands the known function of this protein as well as our knowledge of the details of the novel recombinational switching mechanism for *vlsE* variation.

## Introduction

**FIGURE 1:**
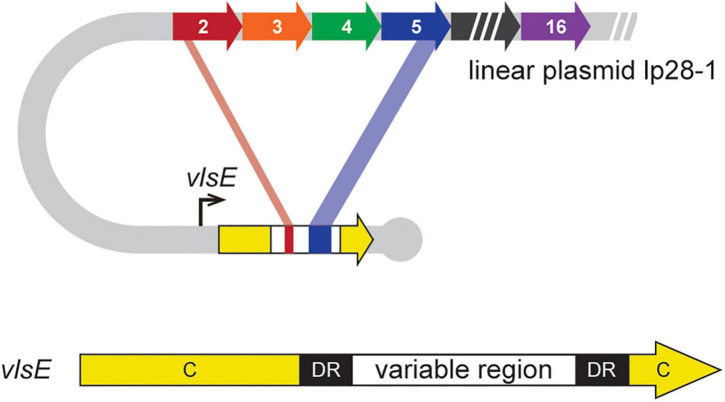
Schematic of DNA rearrangements underlying antigenic variation in *B. burgdorferi.* The *vls* locus is located near the right hairpin telomere on lp28-1 (Zhang et al., 1997). It is composed of an expression locus (*vlsE)* that contains a variable region flanked by 17-bp direct repeats (DR) and constant regions (C). Upstream of *vlsE* are 15 homologous, but different, silent cassettes corresponding to the variable region of *vlsE*, which carry information for unidirectional segmental gene conversion events to generate chimeric VlsE antigens (Norris, 2014; Chaconas et al., 2020). Figure modified from Verhey et al. (2018b).

Obligate parasites have an essential need for ongoing association with a host. In the case of pathogens this translates into the necessity for strategies to maintain a persistent infection. One such pathogenic ruse employed by several types of bacteria, protozoa, and fungi is antigenic variation whereby the pathogen continually changes a major surface protein to generate variants that escape the acquired immune system of the host (Deitsch et al., 2009; Cahoon and Seifert, 2011; Prucca et al., 2011; Vink et al., 2012; Volz et al., 2012; Glover et al., 2013; Horn, 2014; Norris, 2014; McCulloch et al., 2015; Obergfell and Seifert, 2015; Gargantini et al., 2016; Palmer et al., 2016; Devlin et al., 2017; Wahlgren et al., 2017; Sima et al., 2019; Chaconas et al., 2020; Lopez et al., 2021). *Borrelia burgdorferi*, the Lyme disease spirochete encodes a single diversity generating recombination system of this type for the surface-bound lipoprotein VlsE [see Norris (2014) and Chaconas et al. (2020) for recent reviews]. Continual variation of VlsE was first described in and has been characterized primarily in the type strain *B. burgdorferi* B31 (Zhang et al., 1997). In this strain, the *vls* locus is located on the linear plasmid lp28-1 and variability of VlsE is driven by changes at the DNA level of the *vlsE* gene. This occurs through unidirectional segmental gene conversion events (recombinational switching) that transfer information from a series of 15 silent cassettes corresponding to the variable region of *vlsE* (Figure 1), potentially resulting in over ∼10^40^ possible unique VlsE sequences (Verhey et al., 2018a). Recombinational switching does not occur at significant levels during growth in culture, or in ticks, but requires an animal infection to induce the ongoing process, which continues long-term in a mammalian infection (see Norris, 2014; Chaconas et al., 2020). The signal that induces activation of recombination at *vlsE* remains unknown. Antigenic variation of VlsE in *B. burgdorferi* is required for persistent infection of an immunocompetent murine host (Purser and Norris, 2000; Bankhead and Chaconas, 2007; Rogovskyy et al., 2015; Magunda and Bankhead, 2016) and may operate through an epitope-shielding mechanism (Lone and Bankhead, 2020). In the absence of VlsE, infection proceeds at the wild-type level, but spirochete clearance occurs between 12 and 21 days post-infection (Lawrenz et al., 2004; Bankhead and Chaconas, 2007).

Although discovered 25 years ago, the molecular details by which the segmental gene conversion events underlying antigenic variation in *B. burgdorferi* remain largely uncharacterized. Recombinational switching at *vlsE* is homology-driven, but surprisingly, RecA-independent (Liveris et al., 2008; Dresser et al., 2009). The only identified required factor for the reaction is the RuvAB branch migrase (Dresser et al., 2009; Lin et al., 2009), which is shared with the better characterized *pilE* antigenic variation system of *Neisseria gonorrhoeae* (see Chaconas et al., 2020). In spite of the paucity of identified protein factors that mediate recombinational switching, recent advances have been made in characterization of the reaction products through the use of next generation (NG) PacBio long-read sequencing technology. NG sequencing coupled with VAST software (variable antigen sequence tracer), a unique pipeline allowing analysis of large data sets of full-length *vlsE* sequences and inference of silent cassette sources of recombined sequences (Castellanos et al., 2018; Verhey et al., 2018a,b, 2019), has revealed a variety of important new information on switching at *vlsE* [reviewed in Chaconas et al. (2020)].

In this work we have applied our NG sequencing pipeline to the analysis of switching at *vlsE* in several mutant *B. burgdorferi* strains that previously displayed reduced persistence in wild-type C3H/HeN mouse infections. The current study demonstrates a requirement for MutL for recombinational switching, particularly for the putative MutL endonuclease. This is the first report of a role for MutL in bacterial recombination, raises the possibilities that MutL may participate in other recombination activities and expands the known protein factors needed for gene conversion at *vlsE*.

## Results

### Properties of Recombinational Switching at *vlsE* in Various *B. burgdorferi* Replication/Recombination/Repair Mutant Strains

In this work we analyzed recombinational switching at *vlsE* by next generation DNA sequencing in six *B. burgdorferi* B31 5A4 strains carrying mutations in genes encoding the DNA metabolizing proteins *mutL* (DNA mismatch repair), *recJ* (single-strand specific exonuclease), *sbcC* (subunit of SbcCD nuclease), *sbcD* (subunit of SbcCD nuclease), *priA* (primosomal protein N’), and *bbg32* (putative replicative DNA helicase) that previously demonstrated a decrease in persistence at 21 days after infection of wild-type C3H/HeN mice (Dresser et al., 2009). This decrease in persistence is consistent with a decrease in recombinational switching that is more accurately analyzed with NG-sequencing than in our earlier study using small sample sizes and Sanger sequencing. The strains with decreased persistence were also used to infect SCID mice, which lack an acquired immune response. The SCID background removes selective pressure from these mice and allows mutants with decreased switching at *vlsE* to survive in the host by eliminating immune clearance of strains not carrying the most recent VlsE variants. Infection of SCID mice, therefore, provides the best avenue for analysis of recombinational switching without perturbations from acquired immunity. Frozen glycerol cultures of spirochetes recovered from our previous infection of SCID mice (Dresser et al., 2009) were grown from week 0 to week 5. For each strain, genomic DNA was isolated from spirochete cultures obtained at 0 and 5 weeks post-infection. For each gene disruption two *B. burgdorferi* clones were obtained, and two mice were infected per clone. Week 5 genomic DNA was pooled separately for the two replicate mice, from four tissues: bladder, ear, heart, and joint. Week 0 genomic DNA was isolated independently for both clones. A 776 bp amplicon encompassing the *vlsE* variable region and part of the constant region on both sides of the gene was amplified by polymerase chain reaction (PCR) from each sample as previously described (Verhey et al., 2018a), using barcoded primers (Supplementary Table 1) to identify each sample (Supplementary Table 2). The *vlsE* amplicons were sequenced using PacBio long read technology and the data analyzed using our previously reported pipeline and VAST software (Verhey et al., 2018a,b, 2019).

**FIGURE 2:**
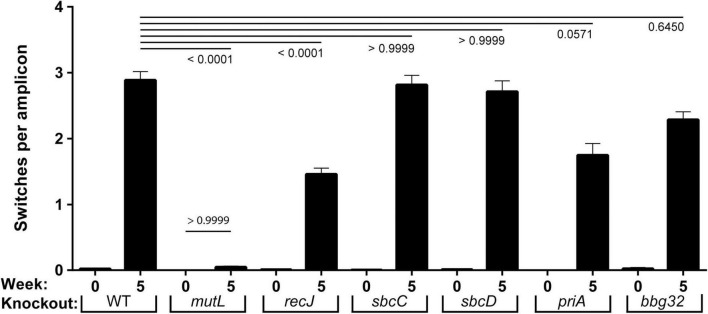
Frequency of *vlsE* switching events in strains with mutant replication/recombination/repair genes. Inferred recombination are shown as a frequency per read events (gene conversion tracts per amplicon) for WT and six mutant strains of *B. burgdorferi* prior to (week 0) and after infection (week 5). Mean ± SEM are shown for each population of full-length *vlsE* amplicon sequences. The *p* values for each mutant versus the wild-type strain at 5 weeks is shown at the top of the graph. The *p* value for comparison of *mutL* at 0 and 5 weeks is shown above the *mutL* bars. The *p* values were determined using Dunn’s multiple comparison test.

The level of recombinational switching at *vlsE* in the SCID mice, where there is no immune selection for any switch variants, is shown in Figure 2. At 5 weeks post-infection there were on average about three switches (distinct patches of sequence changes that match sequences in the silent cassettes) per *vlsE* amplicon in the wild-type strain. As previously noted and as expected for switching at *vlsE* (Verhey et al., 2018a,b, 2019), almost all the observed SNPs were found in the variable regions of *vlsE*, for the wild-type and mutant strains rather than in the constant portions of the gene, which do not contribute to antigenic variation (Supplementary Figure 1).

An unexpected result from the sequencing was the 97% reduction in switching activity to background levels in the *mutL* strain at week 5, which was more severe than our previously estimated 36% decrease using a smaller sample size and a classical DNA sequencing approach (Dresser et al., 2009). This decrease (Figure 2) was highly significant compared to WT (*p* < 0.0001) and was not significantly higher than the background switching at time zero (*p* > 0.9999). In the *recJ* strain we saw a 52% decrease in switching (*p* < 0.0001), similar to our previously reported 47% decrease (Dresser et al., 2009). For *sbcC* and *sbcD*, wild-type levels of switching were displayed. For *priA* a 41% decrease in switching, on the threshold of significance (*p* = 0.057), was observed and for *bbg32* a smaller (28%), but not significant decrease occurred. The *sbc* and *priA* strains were previously reported to undergo switching at wild-type levels with smaller sample size (Dresser et al., 2009).

We also analyzed the change in parental *vlsE* sequence for each mutant between 0 and 5 weeks (Supplementary Figure 2) and found the inverse of what we observed in Figure 2: as expected, those mutants demonstrating higher levels of switching at *vlsE* showed lower levels of the parental *vlsE* sequence at 5 weeks. We also analyzed the number of distinct *vlsE* variants at 5 weeks post-infection as an additional measure of the efficiency of recombinational switching at *vlsE* (Supplementary Figure 3). Again, the mutant strains demonstrating the highest levels of switching in Figure 2, showed the greatest number of switch variants.

**FIGURE 3:**
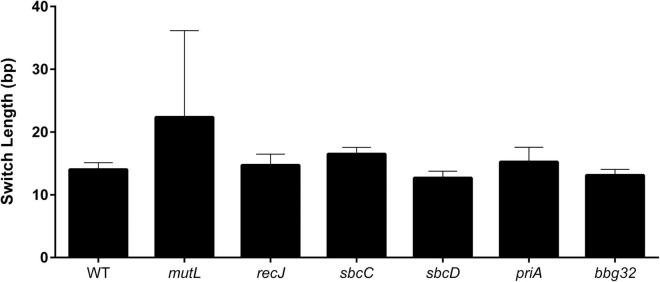
Length of recombination tracts in strains with mutant replication/recombination/repair genes. Recombination tracts were inferred by minimizing the number of recombination tracts required to explain the SNPs of a given read as previously described (Verhey et al., 2018b). The minimum length of each recombination tract is shown as the mean ± SEM for *vlsE* sequences at 5 weeks post-infection.

To further characterize the product of switching in the various mutants, the length of the recombination tract was estimated for each mutant as previously described (Verhey et al., 2018b). The uncorrected mean switch length (Verhey et al., 2018b) for the mutants and the wild-type (Figure 3) agreed well with our previously reported value of 16.4 bp in SCID mice, with the exception of the *mutL* strain, which displayed a somewhat longer (22.4 bp) but statistically insignificant difference in tract length (*p* = 0.55). This may be because only a few switch variants (11) were recovered with the *mutL* strain.

**FIGURE 4:**
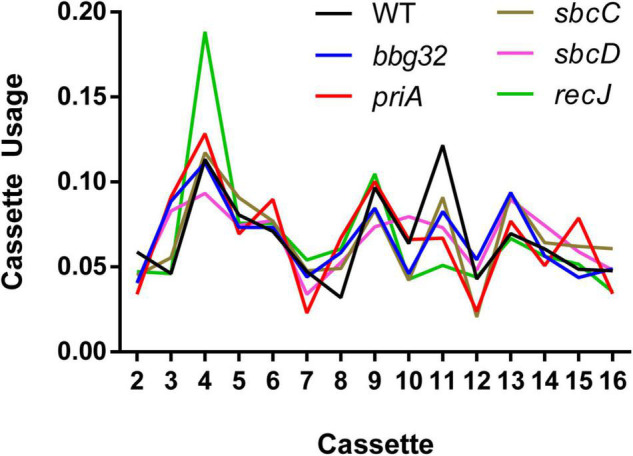
Cassette usage for inferred recombination tracts. For each inferred recombination event, we identified the most likely originating cassette(s). The plot shows the relative usage of each silent cassette as a proportion compared to other cassettes for each mutant strain. Recombination events having *N* possible origins were spread out over all possible silent cassettes with a weight of 1/*N*. The *mutL* gene disruption was omitted due to low switching activity resulting in a small sample size of only 11 recombination events.

**FIGURE 5:**
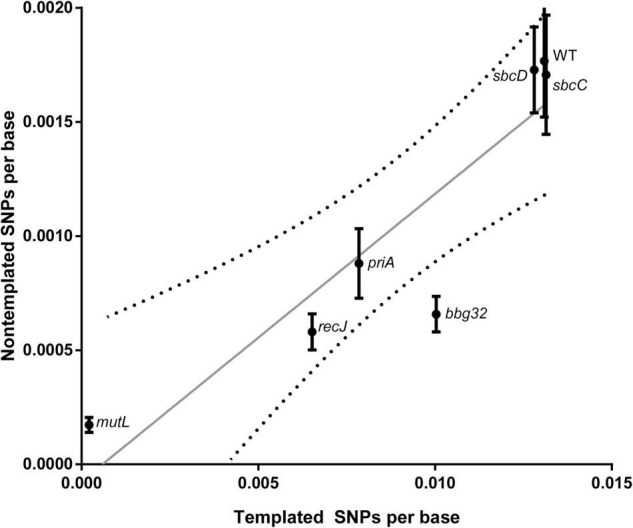
Frequency of non-templated SNPs versus templated SNPs at 5 weeks for WT and each gene disruption mutant. The unit of observation is each base sequenced, and the mean ± SEM is shown. A least-squares regression line and associated 95% confidence bands (dotted lines) are shown.

We then analyzed the silent cassette usage in each of the mutants except *mutL* (Figure 4). There was no significant difference in cassette usage for any of the mutants at any of the 15 silent cassettes. Finally, we analyzed the frequency of non-templated SNPs generated by each mutant (Figure 5). The non-templated changes are generated by error-prone repair and the number is correlated with the level of switch events [26], reminiscent of mutagenic changes associated with mitotic gene conversion in yeast [32]. This complicates a simple analysis to determine whether the various gene disruptions influence the level of non-templated SNPs, since the mutants used show variable levels of templated SNPs. We therefore plotted the frequency of non-templated SNPs against the frequency templated SNPs (Figure 5). Regardless of the recombinational switching level, if the non-templated SNP level per templated switch remains constant for each mutant, the data would be expected to lie on a diagonal line. As shown in the plot, a least squares regression line accommodates the data agree well with a Pearson correlation coefficient, *r* = 0.9086 indicating a similar non-templated SNP frequency per templated SNP for the WT and all the mutants tested, with the exception of *bbg32.* This mutant displayed a 44% decrease from the line of best fit, outside of the 95% confidence limits, suggesting that deletion of the replicative helicase encoded by lp28-2 may result in a less than twofold decrease in non-templated SNP frequency. Because of the multiplicity of helicases and possible pinch-hitting of one helicase for another, assessment of helicase activity in the generation of non-templated SNPs may obscure a possible role in these experiments.

### Generation of MutL Mutants Specifically Defective in Adenosine Triphosphate Binding, Adenosine Triphosphate Hydrolysis, DNA Nicking, or Interaction With the β-Sliding Clamp

**FIGURE 6:**
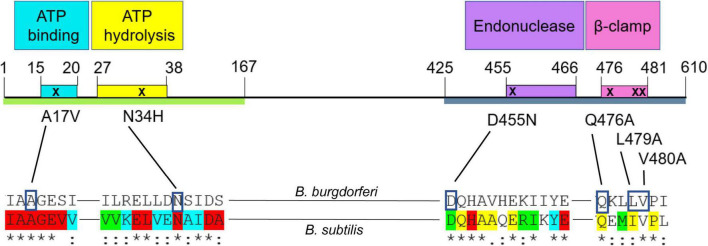
Schematic of *Borrelia burgdorferi* MutL protein and point mutations introduced in this study. The green line represents the N-terminal (ATPase) domain of MutL (Bolz et al., 2012) and the blue one, the C-terminal domain (endonuclease) domain (Pillon et al., 2010, 2011). The colored boxes represent the conserved motifs that confer different activities to the protein (Pillon et al., 2010, 2011; Bolz et al., 2012). Conserved residues in a lineup of about 200 MutL orthologs (Banasik and Sachadyn, 2014) are indicated at the bottom of the figure. The colors show different degrees of conservation, where red indicates an identical status in all the sequences included in the analysis. The blue color indicates only conservative amino acid changes in 100% of the proteins; green indicates only conservative amino acid changes in more than 90% of the sequences analyzed and yellow shows those residues with only conservative changes in 60–90% of the sequences analyzed. The asterisks indicate identical residues between the *B. burgdorferi* and *B. subtilis* alignment, the colons denote amino acids with conserved but non-identical changes.

To determine which functional region(s) of MutL (Fukui et al., 2011; Guarne, 2012; Banasik and Sachadyn, 2014; Guarne and Charbonnier, 2015) are involved in recombinational switching at *vlsE*, we generated chromosomal *mutL* mutants in the endogenous *mutL* locus, with specific changes in motifs of the protein predicted to be involved in adenosine triphosphate (ATP) binding, ATP hydrolysis, endonuclease activity, or in the β-clamp binding motif. The mutations introduced were based upon MutL mutations that affected mismatch repair in the MutL prototype from *Bacillus subtilis* (Pillon et al., 2010, 2011; Bolz et al., 2012). The *B. burgdorferi* mismatch repair system lacks a MutH ortholog as does *B. subtilis*, and the MutL proteins from these species contain an endonuclease motif (Figure 6) believed to be responsible for nicking of mismatched DNA once identified by MutS. In selecting the amino acids in MutL to mutate, we started with previously characterized mutations in *B. subtilis* and chose residues that were identical (or conserved in the case of L479) in *B. burgdorferi* and which demonstrated a phenotype in *B. subtilis* with single amino acid changes in the putative ATP binding, ATP hydrolysis and endonuclease motifs (Supplementary Figure 4). Several residues were changed in the β-clamp binding motif to most effectively neutralize the MutL- β-clamp interaction (Pillon et al., 2010).

**FIGURE 7:**
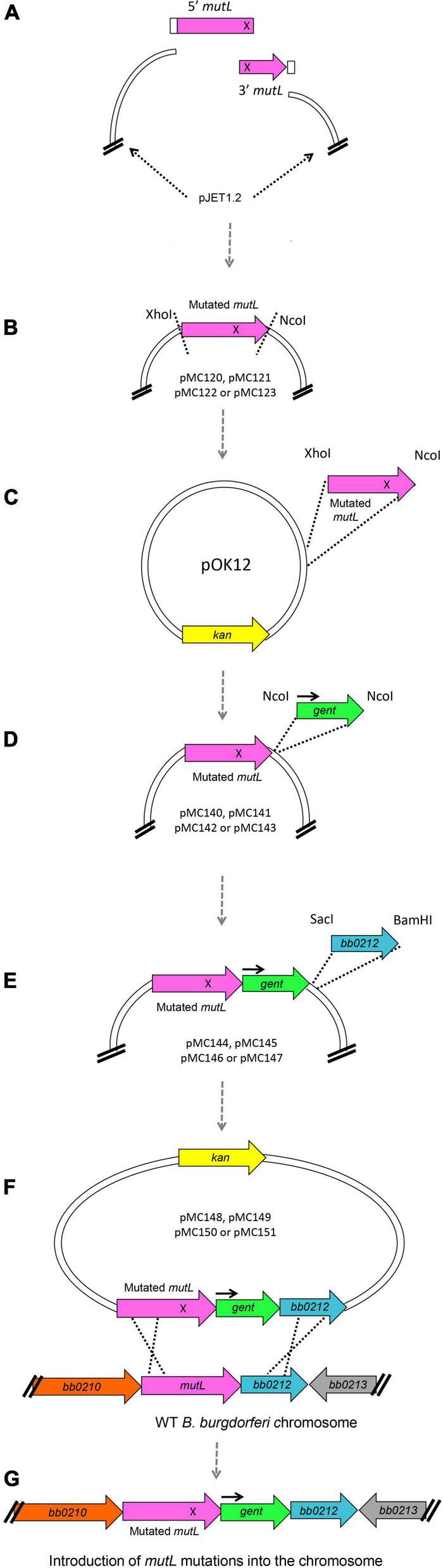
Schematic of plasmid construction and introduction of point mutations into *B. burgdorferi* chromosomal *mutL*. **(A)** To generate the *E. coli* plasmids carrying point mutations in *mutL* we designed a two-insert strategy where two *mutL* double stranded fragments carrying complementary base pair changes (represented as an X) were ligated with each other and to pJET1.2 using the HiFi DNA Assembly mix from NEB. This assembly mix contains a 5′-3′ exonuclease that generates single stranded 3′ overhangs. These overhangs are able to anneal for a high-fidelity polymerase to extend the overhangs and fill in the gaps; finally, a DNA ligase seals the nicks. **(B)** The pJET plasmids containing the mutated versions of *mutL* were then digested with *Xho*I and *Nco*I, and **(C)** the mutated *mutL* inserts were then cloned into pOK12, **(D)** generating the plasmids pMC140, pMC141, pMC142, and pMC143 (see Supplementary Table 3). We then inserted the gentamicin resistance cassette with its own promoter (black arrow) downstream of *mutL* at the *Nco*I site. **(E)** Finally, we cloned the *bb0212* gene downstream of the gent cassette to maintain the natural gene order in the *B. burgdorferi* chromosome. **(F)** Plasmids pMC148, pMC149, pMC150, and pMC151 were used to transform *B. burgdorferi* B31 wild-type clone 5A4 [20] and **(G)** GentR-KanS transformants were further analyzed for double recombination events.

Site-directed mutagenesis of the large 1,830 bp *mutL* gene was not successful in our early attempts, despite our previous experience constructing site directed mutants in the *B. burgdorferi hrpA* gene (Salman-Dilgimen et al., 2013). However, success was achieved using the general strategy shown in Figure 7 to generate chromosomal mutations (see section “Experimental Procedures” for details), which may also be useful for mutating other difficult *B. burgdorferi* genes. To facilitate site-directed mutations in the large (1,830 bp) coding region, we first introduced the point mutations at the complementary ends of double stranded fragments that when annealed would regenerate the full *mutL* gene. These fragments were cloned in pJET (Figure 7A). With the mutated versions in pJET, the resulting *mutL* genes were transferred to pOK12 through conventional cloning (Figures 7B,C). Subsequently the gentamicin resistance cassette was inserted downstream of *mutL* (Figure 7D) followed by the downstream gene *bb0212* (to serve as a recombination target) after the *gent* cassette (Figure 7E). This resulted in the *mutL* donor plasmids pMC148, pMC149, pMC150, and pMC151 (Supplementary Table 3), which were used to transform *B. burgdorferi* B31 5A4 for gentamicin resistance. Clones were further analyzed by PCR for double recombination events (Figure 7F) as described in section “Experimental Procedures” to recover the point mutations in the endogenous *mutL* gene of the *B. burgdorferi* chromosome (Figure 7G).

### Analysis of Recombinational Switching in MutL Mutants in the Motifs for Adenosine Triphosphate Binding, Adenosine Triphosphate Hydrolysis, DNA Nicking, or Interaction With the β-Sliding Clamp

**FIGURE 8:**
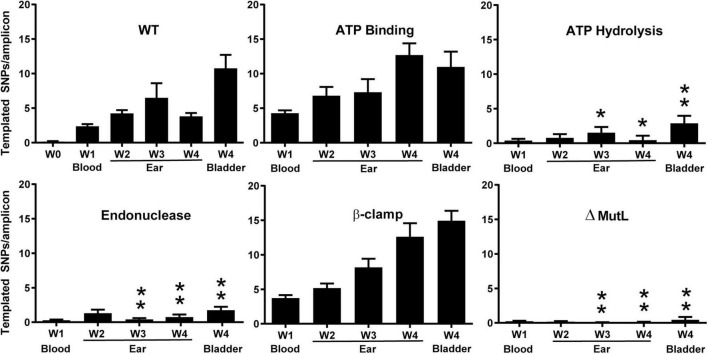
The effect of mutations in MutL on recombinational switching at *vlsE* as measured by the number of templated SNPs per amplicon from NGS sequencing at weeks 1 in mouse blood and at weeks 1–4 in mouse ear and at week 4 in bladder. The wild-type week 0 sample was from spirochetes that were not used to infect mice. *B. burgdorferi* carrying the mutations described in Figure 6 was used to infect SCID mice as described in section “Experimental Procedures.” Mean ± SEM are shown. *P* values were determined for differences between WT and *mutL* mutants in the corresponding tissue type and time. A single asterisk indicates *p* < 0.05 and two asterisks indicated *p* < 0.01. *P* values were determined using two-sample *t*-test with unequal variances.

Following infection of SCID mice with two clones for each mutant (except the ATP hydrolysis mutant), spirochetes were recovered from blood, ear, or bladder. DNA was prepared and *vlsE* gene amplicons were sequenced and analyzed by PacBio long read sequencing and VAST software (Verhey et al., 2018a,b) (see section “Experimental Procedures”). As expected, the wild-type strain showed increasing switching at *vlsE* over time and the *mutL* deletion strain did not show switching above background levels (Figure 8). Interestingly, the putative ATP binding mutant showed no decrease in switching at *vlsE* from the wild-type strain, however, the ATP hydrolysis mutant displayed a significant decrease in recombinational switching. These results were perplexing, because if ATP hydrolysis is required for MutL to promote switching at *vlsE*, then ATP binding should also be required. The simplest proposal for the discrepancy is that the single A17V mutation in *B. burgdorferi* MutL (Figure 6) is not sufficient to disrupt ATP binding, even though it does so in the orthologous *B. subtilis* MutL. Another possibility is that the ATP-off (or ADP-on) state is active in *vlsE* switching. A concern with the ATP binding versus hydrolysis comparison was that only a single mutant clone in the ATP hydrolysis motif was used in the data shown in Figure 8. We therefore repeated the SCID mouse infection with two additional mutant clones and analyzed the results using classical sequencing of independent switch variants (Dresser et al., 2009). The mutant clone used for Figure 8 and the two additional ATP hydrolysis mutant clones all demonstrated a dramatic decrease in *vlsE* switching (Supplementary Figure 5), confirming our initial result shown in Figure 8.

The most interesting result from the analysis of the *mutL* mutants was the apparent requirement of the putative MutL endonuclease activity for recombinational switching at *vlsE*. A dramatic and significant decrease in templated SNPs was observed with the D455N endonuclease mutant. This is a conversion of the aspartic acid to the corresponding amide that is not expected to affect the three-dimensional structure of the protein but is expected to disrupt the metal ion binding required for hydrolytic activity, suggesting that the DNA endonuclease activity is required for switching at *vlsE.* Finally, the triple mutation in the β-clamp binding motif of MutL did not reduce the level of recombinational switching.

## Discussion

### MutL Is Required and RecJ Appears to Play a Role in *vlsE* Gene Conversion

The MutL protein (Fukui et al., 2011; Guarne, 2012; Banasik and Sachadyn, 2014; Guarne and Charbonnier, 2015) and its orthologs are involved in mismatch repair (Iyer et al., 2006; Modrich, 2006, 2016), an important process in all organisms whereby mis-incorporated bases from replication errors are removed and the original DNA sequence is restored. The bacterial MutS protein is responsible for recognizing mismatches and in organisms that encode it, MutH cleaves the DNA backbone for subsequent processing. In organisms lacking a MutH ortholog, MutL encodes the endonuclease activity to nick the DNA for entry of an exonuclease and removal of the area containing the mismatched base, preparing the area for resynthesis and correction. The phenotype of a *mutL* mutant in prokaryotes is a dramatic increase in mutation frequency (Schaaper, 1993). This is expanded in eukaryotes by a dramatic increase in cancers, such as in Lynch syndrome (Olave and Graham, 2021). The pronounced reduction in recombinational switching to background levels in the *vlsE* gene in the absence of MutL (Figure 2 and Supplementary Figures 2, 3) was surprising, especially because deletion of key interacting partner, MutS, did not affect either the switching frequency or the infectivity of *mutS* spirochetes in immunocompetent mice (Dresser et al., 2009). Taken together, these results point to an unexpected role for MutL that is outside its usual mismatch repair function (since MutS is not involved) and raises the possibility that MutL may be directed to *vlsE*, the silent cassettes, or a synapse between them, by a yet unidentified protein. This work reports for the first time a function for MutL in bacterial DNA recombination.

In addition to an important role for MutL, the absence of RecJ (Lovett, 2011; Cheng et al., 2016) resulted in a significant 50% decrease in switching in *vlsE* (Figure 2 and Supplementary Figures 2, 3). It is noteworthy that the RecJ 5′ to 3′ exonuclease often functions at nicks generated by MutL (Fukui et al., 2011; Guarne, 2012) suggesting that their paired activity may be important for recombinational switching. The failure of the *recJ* mutant to completely block gene conversion at *vlsE* is not surprising since RecJ shares overlapping functions with other cellular exonucleases (Lovett, 2011) that might pinch-hit in its absence.

Finally, disruption of the PriA replication restart protein (Michel et al., 2018; Windgassen et al., 2018) resulted in a 41% decrease in switching (Figure 1), on the threshold of significance (*p* = 0.057). However, changes in parental DNA sequences (Supplementary Figure 2) or distinct variants (Supplementary Figure 3), relative to wild-type did not vary significantly, leaving a possible role for the PriA helicase as an open question at this time.

### The Putative MutL Endonuclease Is Required for *vlsE* Gene Conversion

To further probe the role of MutL in recombinational switching at *vlsE*, site directed mutations were generated in the putative ATP binding, ATP hydrolysis, endonuclease, and β-clamp binding motifs previously identified and characterized in *B. subtilis* (Figure 6). Interestingly, mutations in either the ATP binding or β-clamp binding motifs did not display a decrease in recombinational switching at *vlsE.* In contrast, mutagenesis of both the endonuclease and ATP hydrolysis motifs resulted in significant dramatic decreases in gene conversion (Figure 8). Our favored interpretation of the discrepancy in the ATP-interacting motifs is that the conservative change in the ATP binding motif of the A17V mutant is ineffectual at blocking ATP binding. Another possibility is that the ATP-off (or ADP-on) state is active in *vlsE* switching. However, we also cannot rule out that the single amino acid change in the hydrolysis mutant (N34H) is disruptive to the overall protein structure, thereby abrogating gene conversion by an indirect effect rather than specifically blocking ATPase activity. This possibility seems less likely to us in view of the demonstrated specificity of the mutation in *B. subtilis* MutL. The lack of an effect of the ATP binding mutant in contrast to the dramatic phenotype of the ATP hydrolysis mutant will require further analysis by genetic and/or biochemical approaches not currently available *in vivo* or *in vitro* for *B. burgdorferi* MutL.

The endonuclease activity of MutL (in bacteria lacking MutH) unlocks the double helix for exonucleolytic removal of mis-incorporated bases. *B. burgdorferi* lacks a MutH ortholog and its MutL N-terminal domain carries a clearly recognizable endonuclease motif (Figure 6). Substitution of aspartic acid 455 with an asparagine removed an essential catalytic residue based upon structure and function of the *B. subtilis* enzyme (Pillon et al., 2010). The dramatic decrease in gene conversion from the substitution of the acid with an amide (change of the hydroxyl group to an amino group) at position 455 strongly suggests that the putative endonuclease activity is required for gene conversion at *vlsE* (Figure 8). This type of mutagenic approach in the metal ion binding site of many hydrolytic enzymes has been used to identify active site residues. The ATPase region in the N-terminal domain of MutL (Ban et al., 1999; Fukui et al., 2011) has a role in modulating the endonuclease structure and function in the C-terminal domain of MutL (Pillon et al., 2010; Yamamoto et al., 2011; Bolz et al., 2012) and we speculate that the *B. burgdorferi* ATP hydrolysis mutant manifests its phenotypic effect by blocking the endonuclease activity.

Finally, mutation of three residues in the β-clamp binding motif (Figure 6) did not display any negative effects on gene conversion at *vlsE* (Figure 8). The β-clamp can modulate the endonuclease activity of *B. subtilis* MutL (Pillon et al., 2011, 2015; Almawi et al., 2019) and the results suggest (if the mutations disrupt MutL- β-clamp interactions) that the β-clamp does not play a role in recombinational switching at *vlsE.* The efficacy of this triple MutL mutant in abrogating β-clamp interactions will need to be confirmed by genetic or biochemical approaches.

### Role of the Putative MutL Endonuclease in Recombinational Switching

Endonuclease activities are common initiators of DNA recombination reactions. The generation of double strand breaks (Haber, 2018; Wright et al., 2018; Li et al., 2019) or single strand discontinuities (Maizels and Davis, 2018) can promote recombination either by providing a DNA end for strand invasion, allowing an entry point for an exonuclease or by cleaving branched junctions to resolve recombination intermediates, as is the case in eukaryotic meiotic recombination. The MutL endonuclease could potentially be involved in either the initiation or resolution steps of recombination or both. The endonuclease activity of MutL is non-specific and has not been reported to recognize any specific sequence or DNA lesion (Fukui et al., 2011; Guarne, 2012). How then might MutL be recruited to the *vlsE* locus to participate in generating segmental gene conversion events? MutL has been reported to interact with several other proteins including MutS, UvrD and the DNA polymerase β-clamp. One option for targeting is through protein-protein interactions (Friedhoff et al., 2016), although this still leaves open the question of how the *vls* locus is recognized in the first place. A second possibility might be a direct recruitment whereby MutL directly recognizes the *vls* locus. We have previously reported an exceedingly high preponderance of strand-specific G-runs of 3 nucleotides or longer in *vlsE* and in the silent cassette coding strands (Walia and Chaconas, 2013; Chaconas et al., 2020). It is tempting to speculate that assembly of transient G-quadruplex structures from the multitude of G-runs might provide a target for recruitment of MutL, which through its endonuclease could generate a free DNA end for strand invasion to initiate a recombination reaction. A recent report of specific binding of *Escherichia coli* MutL to G4 DNA (Pavlova et al., 2020) suggests that this possibility is worth further investigation by biochemical characterization of the *B. burgdorferi* MutL protein.

Although a role for MutL in recombination has not been previously reported in bacteria, several reports of a role for MutL orthologs in recombination in higher organisms have (Argueso et al., 2004; Nishant et al., 2008; Zakharyevich et al., 2012; Ranjha et al., 2014; Rogacheva et al., 2014; Duroc et al., 2017; Manhart et al., 2017; Toledo et al., 2019; Cannavo et al., 2020; Kulkarni et al., 2020; Rahman et al., 2020). Of particular interest is the consistent implication of the endonuclease activity of MutL orthologs at a late step of homologous recombination as a double Holliday junction resolving enzyme that is activated by PCNA (Kulkarni et al., 2020), the eukaryotic equivalent of the bacterial β-clamp protein. Interestingly, our triple substitution (Figure 6) in the β-clamp interacting domain of MutL displayed recombinational switching at wild-type levels (Figure 8) suggesting the possibility that perhaps the endonuclease activity may not be involved in the junction resolution step at *vlsE*.

Previously we and the Norris lab (Dresser et al., 2009; Lin et al., 2009) had shown that the RuvAB complex, which can branch-migrate Holliday junctions, was a key factor in *vlsE* diversification. In many bacteria, RuvAB acts in conjunction with the Holliday junction resolvase, RuvC; but a RuvC ortholog is apparently absent from *B. burgdorferi*. It is possible that another structure-specific endonuclease could act in place of RuvC, and indeed, as noted above, in eukaryotes, the MutL homolog complex Mlh1-Mlh3, has been shown to cleave such branched structures (Argueso et al., 2004; Nishant et al., 2008; Zakharyevich et al., 2012; Ranjha et al., 2014; Rogacheva et al., 2014; Duroc et al., 2017; Manhart et al., 2017; Toledo et al., 2019; Cannavo et al., 2020; Kulkarni et al., 2020; Rahman et al., 2020).

Another possibility is that recombinogenic lesions are generated by the processing of site-specific cytidine deaminations, and depend on MutL. The *vls* gene arrangement in *B. burgdorferi* is strikingly similar to the immunoglobulin genes in chickens, where a single V(D)J segment is diversified by gene conversions from an array of adjacent pseudogenes. In the avian system, these gene conversions are initiated by chromosome breaks created by the mismatch repair-dependent processing of dU::G base pairs that are created by the activation-induced cytidine deaminase (AID; Di Noia and Neuberger, 2007; Arakawa and Buerstedde, 2009; Lanning and Knight, 2015).

The required role of MutL for recombination in a recombination-driven antigenic variation pathway is uniquely reported here for *B. burgdorferi.* This, plus additional unique features of this reaction such as its RecA independence (Liveris et al., 2008; Dresser et al., 2009) and the dispensability of a variety of other proteins required by the well-characterized *N. gonorrhoeae* system for antigenic variation (Obergfell and Seifert, 2015; Chaconas et al., 2020), make further studies on gene conversion in *B. burgdorferi* an exciting frontier, ripe for further study and development of biochemical and genetic analyses.

## Experimental Procedures

### Construction of Plasmids Carrying *mutL* Mutations

All strains and plasmids used in this study are noted in Supplementary Table 3 and were propagated in BSK-II prepared in-house and supplemented with 6% rabbit serum (Barbour, 1984). Strains were stored at −80°C in medium containing 20% glycerol. *B. burgdorferi* constructs were made in the B31 5A4 strain (Purser and Norris, 2000). Gene disruption mutants were generated by allelic exchange as previously described (Dresser et al., 2009). To generate mutations in various *B. burgdorferi* MutL motifs (Supplementary Table 3), we introduced point mutations in the regions for ATP binding, ATP hydrolysis activity, the endonuclease activity and the region interacting with the DNA polymerase β-clamp (Figure 6, Supplementary Table 4, and Supplementary Figure 4). As a general strategy, primers were designed to amplify the full *mutL* (or full *mutL* plus part of the upstream gene, depending on the domain to be mutated) in two parts with overlapping sequences so they could be joined without “scars” other than the point mutations. The point mutations were introduced at the overlapping sequences that the primers carry (Supplementary Table 4 and Figure 7). The primer combinations are as follows: for the ATP Binding site we used primers B3049 and B3050 (left side, 643 bp), and B3051 and B3052 (right side, 1,824 bp), for the ATP hydrolysis KO mutant we used primers B3049 and B3053 (left side, 689 bp), and B3054 and B3052 (right side, 1,778 bp). For the endonuclease point mutation we used primers B3055 and B3056 (left side, 1,117 bp), and B3057 and B3052 (right side, 512 bp). Finally, to mutate the β-clamp binding domain we used primers B3055 and B3058 (left side, 1,193 bp), and B3059 and B3052 (right side, 433 bp). The 5′ and 3′ ends of *mutL* also have overlapping sequences to pJET1.2 (NEB) so they could be sub-cloned first. Amplification conditions for the *mutL* PCRs are as follows: a denaturation step at 95°C for 2 min, followed by 30 cycles at 95°C for 15 s, 50 or 54°C for 15 s and 68°C for 60 s, finished by a final extension cycle at 68°C for 5 min. Phusion High Fidelity DNA Polymerase from NEB was used for all the PCRs described here. PCR products were gel purified when they displayed non-specific bands (Zymoclean Gel DNA recovery kit) or cleaned with a PCR column (Qiagen PCR purification kit). Ligations were performed using the NEBuilder HiFi DNA Assembly master mix by adding 0.14 pmols per insert (right and left sides of *mutL* for each domain to be mutated) and 0.07 pmols of pJET1.2 (NEB) in a final volume of 20 μL. Ligations were incubated at 50°C for 15 min and 3 μL were used to transform chemically competent DH5-α cells. Candidates were analyzed by digesting the plasmid DNA with *Bgl*II (NEB) for 2 h at 37°C. Those that had the expected band sizes were then sequenced to confirm the presence of the point mutations. Sanger sequencing was done by using the pJET1.2 Forward and Reverse primers using a 60°C annealing temperature.

Subsequently, the full *mutL* gene was transferred to the vector pOK12 (Vieira and Messing, 1991) which is unable to replicate in *B. burgdorferi*. To do this, we digested the pOK12 and pJET::*mutL* constructs with *Xho*I and *Nco*I with 10 units of each enzyme overnight. Bands were purified from 1% agarose gels (Zymoclean Gel DNA recovery kit) and ligated with T4 DNA ligase from NEB for 2 h at room temperature. We used 50 ng of pOK12 with a 1:1 vector:insert molar ratio. 5 μL of the ligation reactions were used to transform competent DH5-α cells. Candidate colonies were analyzed through digestion patterns with *Xho*I and *Nco*I. In this way we generated plasmids pMC140, pMC141, pMC142, and pMC143 (Supplementary Table 3). The next step was to clone the gentamicin cassette downstream of point mutated *mutL* in these constructs. The *gent* cassette was amplified with oligos B3061 and B3094, introducing *Nco*I sites on both sides (Supplementary Table 4), by 30 cycles at 95°C for 10 s, 60°C for 10 s, and 72°C for 25 s, and followed by a final extension cycle at 72°C for 5 min. The gentamicin product and plasmids pMC140, pMC141, pMC142, and pMC143 were digested overnight with *Nco*I and gel purified. Ligations were performed in molar ratios 1:5 vector:insert with T4 DNA ligase at 16°C overnight. 5 μL of the reactions were used to transform competent DH5-α. Since the gentamicin cassette could be cloned in either orientation, we selected those that had it in the same direction as *mutL*. To this end we used the restriction enzyme *Nde*I and incubated the reactions for 2 h at 37°C. We called the new plasmids: pMC144, pMC145, pMC146, and pMC147 (Supplementary Table 3).

The final step in the construction of the domain knock-out vectors was to clone *bb0212* downstream of the gentamicin cassette; this is the gene located downstream of *mutL* in the wild type configuration. *bb0212* was amplified with oligos B3101 and B3102 (Supplementary Table 4), which contained the *Sac*I and *Bam*HI restriction sites, respectively. Amplification conditions were done with a first denaturation step at 95°C for 2 min, followed by 30 cycles at 95°C for 10 s, 55°C for 10 s, and 68°C for 25 s, finished with a final extension cycle at 68°C for 5 min. Plasmids pMC144, pMC145, pMC146, and pMC147 and the *bb0212* gene were digested with *Sac*I and *Bam*HI overnight and purified from 1% agarose gels. Ligations were performed at molar ratios of 1:3 vector:inserts and incubated 2 h at room temperature. 5 μL was used to transform competent DH5-α cells and candidate colony plasmid DNAs were digested with either *Bam*HI or *Spe*I. Those clones that produced the expected digestion patterns were then sent sequenced to verify the presence of the desired mutations and the integrity of the remainder of the *mutL* gene. The oligos used to sequence *mutL* were: B3107, B3108, B3109, B3110, and B3052 (Supplementary Table 4). Final plasmids were named plasmids pMC148, pMC149, pMC150, and pMC151.

### Introduction of *mutL* Domain Mutants Into the *B. burgdorferi* Chromosome

Allelic exchange was performed as previously described (Samuels et al., 2018). The construction strategy used in this work is outlined in Figure 7. The gene of interest was amplified and cloned into a vector unable to replicate in *B burgdorferi*. Then an antibiotic cassette was inserted in the middle of the gene to interrupt it, but conserving homologous regions flanking the antibiotic cassette that serve as recombination targets. This construct was used to transform *B. burgdorferi* strain B31 5A4 (Purser and Norris, 2000; see Supplementary Table 1). For allelic exchange, 50 μg of plasmids pMC148, pMC149, pMC150 and 40 μg of pMC151 were used in the transformations. To screen for transformants that had acquired the mutations, we designed primers that contain the point mutations at the 3′ end of the relevant primers such that amplification occurred only if the mutation had been introduced but with the wild-type gene. Primers used to screen the ATP binding and ATP hydrolysis domain mutants were B3113 and B3107, and B3107 and B3114, respectively. The PCR program used for them was a two-step PCR for 30 cycles at 95°C for 10 s and 68°C for 20 s of annealing/extension. To screen for the endonuclease mutation, we used primers B3115 and B3117 with an annealing temperature of 58°C, while the denaturing and extension steps were the same as for the ATP binding and hydrolysis mutants. Finally, for the β-clamp binding site mutation, we used primers B3116 and B3117 at an annealing temperature of 50°C. All PCRs were carried out with Phusion polymerase from NEB. After identifying at least five candidates per construct, we grew them to saturation and purified the genomic DNA with two extractions of phenol-chloroform-isoamyl alcohol (25:24:1) and precipitation with isopropanol. Once the DNA was purified we did PCRs to screen for the presence of gentamicin and the absence of the kanamycin cassette by using the following conditions: 2 min at 95°C, followed by 30 cycles of 10 s at 95°C, 10 s at 50°C and 30 s at 68°C, and finally one cycle of 5 min at 68°C. PCR bands including the point mutations were amplified and sent for Sanger sequencing to confirm that the mutations were present. To this end we used oligos B3107 and B3122 for the ATP binding and ATP hydrolysis mutants, and B3109 and B3124 for the endonuclease and the β-clamp binding domain mutants. PCR conditions were 2 min at 95°C, followed by 30 cycles of 10 s at 95°C, 10 s at 60°C, and 20 s at 68°C, to finalize with one cycle of 5 min at 68°C. The same oligos were used to sequence the bands at 60°C.

Further characterization was performed to ascertain that our mutants were not merodiploid where only one recombination event occurred, instead of two, producing a cointegrate rather than an allelic replacement. To analyze possible merodiploidy we used the primers B3102 and B3110 where a 2,176 bp band would indicate an allelic replacement, while a 1,472 bp band would reveal the presence of wild-type copies of *mutL*. The PCRs were performed by incubating at 2 min at 95°C, followed by 30 cycles of 10 s at 95°C, 10 s at 60°C, and 60 s at 68°C, and finalized with one cycle of 5 min at 68°C. For the β-clamp binding site domain mutation, another primer set was used under the same conditions described above (B3110 and B3128).

Finally, we assessed plasmid content using a multiplex PCR assay (Bunikis et al., 2011) for all our mutant clones to confirm that they had not lost essential plasmids for infectivity and persistence. PCR conditions were as follows: one cycle to denature the DNA at 95°C for 2 min, then 30 cycles at 95°C for 30 s, 60°C for 60 s, 68°C for 60 s, and a final cycle at 68°C for 5 min.

### Mouse Infections and Isolation of *B. burgdorferi* Genomic DNA

Mouse infections for Figures 2–5 were previously described (Dresser et al., 2009). Spirochetes were cultured from the frozen glycerol stocks from the SCID mouse infections from week 0 to week 5 (Dresser et al., 2009), and genomic DNA was isolated. For each gene disruption two *B. burgdorferi* clones were obtained, and two mice were infected per clone. Week 5 genomic DNA was pooled from spirochete cultures from four tissues: bladder, ear, heart, and joint. For the more recent *mutL* mutant infections, a total of 24 Fox Chase SCID male mice (C.B-17 SCID) were purchased from Charles River Laboratories. Two independent clones of each construct were individually grown to infect mice with 10^4^ spirochetes in exponential growth phase in BSK-II medium. Additionally, two more clones belonging to the parental isogenic 5A4 strain and two clones belonging to the full *mutL* knock out were also used as positive and negative controls, respectively. Two mice were infected per clone, for a total of four mice per mutant (Supplementary Table 2). The inoculation was done by subcutaneous injection into the backs of mice at 4–6 weeks of age. DietGel^®^ Boost (ClearH2O, Westbrook, ME, United States) was added to the cages as a supplement to help maintain weight. Tissue samples were collected at week 1 (50 μl of blood from the tail vein), week 2 and week 3 (ear punches), and week 4 post-infection (ear punches and bladder). Each of these samples was grown in BSK-II with 6% rabbit serum and antibiotics (0.02 mg ml^–1^ phosphomycin, 0.05 mg ml^–1^ rifampicin, and 2.5 μg ml^–1^ amphotericin B) to which *B. burgdorferi* is naturally resistant. Genomic DNA was purified from 8 ml cultures by phenol-chloroform extraction followed by isopropanol precipitation (Verhey et al., 2018a).

### *vlsE* Polymerase Chain Reaction Amplification and Sequencing

The barcoded primers used in the present work were previously described in Verhey et al. (2018a) and Supplementary Table 1. The primers amplify a 776 bp band from *vlsE*, and contain barcodes that allow sample identification. We used a paired barcode scheme, where the combination of the forward and the reverse primer uniquely identified each sample (Supplementary Table 2). PCRs were performed for 30 cycles either with a three-step (Verhey et al., 2018a) or two-step (Dresser et al., 2009) cycle as previously described. Phusion Polymerase from New England Biolabs (NEB) was used for all amplifications. PCR products were quantified by agarose gel densitometry using Image-J, with standard curves made from DNA ladders from NEB following staining with ethidium bromide. Following quantification, samples were pooled, concentrated on a Qiagen QIAquick PCR Purification kit (Qiagen), run in an agarose gel and gel-purified without staining using either the Qiagen or Monarch (NEB) gel extraction kit. The recovered material was quantified by ethidium bromide staining and gel densitometry. The amplicon mixture was sequenced by PacBio long-read technology using the RS II instrument at Génome Québec Innovation Centre and three SMRT cells (Figures 2–5) and using the Sequel II instrument and one SMRT cell for Figure 8. Sanger sequencing of *vlsE* was performed as previously reported (Dresser et al., 2009).

### Bioinformatics

PacBio sequencing data was processed from raw BAM files obtained from Génome Québec. To generate DNA strand-specific circular consensus sequences in FASTQ format, we used CCS v6.0.0 (Pacific Biosciences)^1^, and specified the –by-strand option. For Figures 2–5, we additionally filtered consensus reads with a minimum read quality of 0.99999 (equivalent to Q50), but set the –min-passes filter to 0 and the –min-snr filter to 0. For Figure 8 we specified a minimum read quality of 0.99975 (equivalent to Q36) but retained the default –min-passes filter of 3 and the –min-snr filter of 2.5. Sequences were demultiplexed using fdemux^2^, which detects unique asymmetric barcode pairs from PacBio reads. To maintain highly accurate demultiplexing, exactly two barcodes in the expected orientation were required, and an edit distance of 2 was specified to allow for some errors. Since any one barcode is at least 6 edits away from another, this should prevent false positives in demultiplexing.

Sequences were then trimmed to include 5′ and 3′ 16-mer flanking sequences which are invariant in the dataset due to their location in the constant region and inclusion in the PCR primers. These sequences were CGACGGGGAAACCAGA for the 5′ end of the amplicon and AGGCTGCTAGTAAAGA for the 3′ end.

Sequence data was then analyzed using our previously reported Variable Antigen Sequence Tracer software (Verhey et al., 2018a). Statistics were computed using GraphPad Prism 6.0.

## Accession Numbers

PacBio sequencing data is available in the NCBI Sequence Read Archive (SRA) at https://www.ncbi.nlm.nih.gov/sra: DNA replication and repair mutant sequencing, SRR18680908; MutL mutant sequencing, SRR18680907.

## Data Availability Statement

All relevant data are within the manuscript and Supplementary Material, further inquiries can be directed to the corresponding author.

## Ethics Statement

All animal experimentation was carried out in accordance with the principles outlined in the most recent policies and Guide to the Care and Use of Experimental Animals by the Canadian Council on Animal Care. The animal protocols (AC16-0068 and AC20-0031) were approved by the Animal Care Committee of the University of Calgary.

## Author Contributions

GC, TV, and MC: conception or design of the work. TV, MC, and MG: data collection. GC and TV: data analysis and interpretation. GC, TV, MC, and MG: drafting the article and final approval of the version to be published. GC: critical revision of the manuscript. All authors contributed to the article and approved the submitted version.

## Conflict of Interest

The authors declare that the research was conducted in the absence of any commercial or financial relationships that could be construed as a potential conflict of interest.

## Publisher’s Note

All claims expressed in this article are solely those of the authors and do not necessarily represent those of their affiliated organizations, or those of the publisher, the editors and the reviewers. Any product that may be evaluated in this article, or claim that may be made by its manufacturer, is not guaranteed or endorsed by the publisher.

## Footnotes

1
https://github.com/PacificBiosciences/ccs

2
https://github.com/verheytb/fdemux
