# Supplementary material for: The Putative Endonuclease Activity of MutL Is Required for the Segmental Gene Conversion Events That Drive Antigenic Variation of the Lyme Disease Spirochete
Source: Front Microbiol. 2022 May 19;13:888494. doi: 10.3389/fmicb.2022.888494 (PMC9159922; doi:10.3389/fmicb.2022.888494)
Supplement: Supplementary file 1 [file Data_Sheet_1.PDF]

Table S1: Barcoded PCR Primers used in this study

**Name**	**Direction**	**Barcode**	**Primer Sequence** (5' to 3', barcode **TARGET**)
B2736	Forward	1	TCAGACGATGCGTCAT**GCGATATAAGTAGTACGACGGGGAAACCAG**
B2737	Forward	2	CTATACATGACTCTGC**GCGATATAAGTAGTACGACGGGGAAACCAG**
B2738	Forward	3	TACTAGAGTAGCACTC**GCGATATAAGTAGTACGACGGGGAAACCAG**
B2739	Forward	4	TGTGTATCAGTACATG**GCGATATAAGTAGTACGACGGGGAAACCAG**
B2740	Forward	5	ACACGCATGACACACT**GCGATATAAGTAGTACGACGGGGAAACCAG**
B2741	Forward	6	GATCTCTACTATATGC**GCGATATAAGTAGTACGACGGGGAAACCAG**
B2742	Forward	7	ACAGTCTATACTGCTG**GCGATATAAGTAGTACGACGGGGAAACCAG**
B2743	Forward	8	ATGATGTGCTACATCT**GCGATATAAGTAGTACGACGGGGAAACCAG**
B2744	Forward	9	CTGCGTGCTCTACGAC**GCGATATAAGTAGTACGACGGGGAAACCAG**
B2971	Forward	10	GCGCGATACGATGACT**GCGATATAAGTAGTACGACGGGGAAACCAG**
B2972	Forward	11	CGCGCTCAGCTGATCG**GCGATATAAGTAGTACGACGGGGAAACCAG**
B2973	Forward	12	GCGCACGCACTACAGA**GCGATATAAGTAGTACGACGGGGAAACCAG**
B2974	Forward	13	ACACTGACGTCGCGAC**GCGATATAAGTAGTACGACGGGGAAACCAG**
B2975	Forward	14	CGTCTATATACGTATA**GCGATATAAGTAGTACGACGGGGAAACCAG**
B2976	Forward	15	ATAGAGACTCAGAGCT**GCGATATAAGTAGTACGACGGGGAAACCAG**
B2977	Forward	16	TAGATGCGAGAGTAGA**GCGATATAAGTAGTACGACGGGGAAACCAG**
B2978	Forward	17	CATAGCGACTATCGTG**GCGATATAAGTAGTACGACGGGGAAACCAG**
B2979	Forward	18	CATCACTACGCTAGAT**GCGATATAAGTAGTACGACGGGGAAACCAG**
B2980	Forward	19	CGCATCTGTGCATGCA**GCGATATAAGTAGTACGACGGGGAAACCAG**
B2981	Forward	20	TATGTGATCGTCTCTC**GCGATATAAGTAGTACGACGGGGAAACCAG**
B2982	Forward	21	GTACACGCTGTGACTA**GCGATATAAGTAGTACGACGGGGAAACCAG**
B2983	Forward	22	CGTGTCGCGCATATCT**GCGATATAAGTAGTACGACGGGGAAACCAG**
B2984	Forward	23	ATATCAGTCATGCATA**GCGATATAAGTAGTACGACGGGGAAACCAG**
B2985	Forward	24	GAGATCGACAGTCTCG**GCGATATAAGTAGTACGACGGGGAAACCAG**
B2986	Forward	25	CACGCACACACGCGCG**GCGATATAAGTAGTACGACGGGGAAACCAG**
B2987	Forward	26	CGAGCACGCGCGTGTG**GCGATATAAGTAGTACGACGGGGAAACCAG**
B2988	Forward	27	GTAGTCTCGCACAGAT**GCGATATAAGTAGTACGACGGGGAAACCAG**
B2989	Forward	28	GAGACTCTGTGCGCGT**GCGATATAAGTAGTACGACGGGGAAACCAG**
B2990	Forward	29	GCTCGACTGTGAGAGA**GCGATATAAGTAGTACGACGGGGAAACCAG**
B2991	Forward	30	AGAGATGTGTGATGAC**GCGATATAAGTAGTACGACGGGGAAACCAG**
B2992	Forward	31	TACGACTACATATCAG**GCGATATAAGTAGTACGACGGGGAAACCAG**
B2993	Forward	32	TATCTCTGTAGAGTCT**GCGATATAAGTAGTACGACGGGGAAACCAG**
B2994	Forward	33	AGAGAGAGACATGCGC**GCGATATAAGTAGTACGACGGGGAAACCAG**
B2995	Forward	34	ACTCTCGCTCTGTAGA**GCGATATAAGTAGTACGACGGGGAAACCAG**
B2996	Forward	35	TCTATGTCTCAGTAGT**GCGATATAAGTAGTACGACGGGGAAACCAG**
B2997	Forward	36	GCGTATATCTCATGCG**GCGATATAAGTAGTACGACGGGGAAACCAG**
B2998	Forward	37	GTGCGTATGTCGCTAC**GCGATATAAGTAGTACGACGGGGAAACCAG**
B2999	Forward	38	TGCTCGCAGTATCACA**GCGATATAAGTAGTACGACGGGGAAACCAG**
B3000	Reverse	1	ATGACGCATCGTCTGA**CAAGGCAGGAGGTGTTTCTTTACTAGCAGC**
B3001	Reverse	2	GCAGAGTCATGTATAG**CAAGGCAGGAGGTGTTTCTTTACTAGCAGC**

B3002	Reverse	3	GAGTGCTACTCTAGTA**CAAGGCAGGAGGTGTTTCTTTACTAGCAGC**
B3003	Reverse	4	CATGTACTGATACACA**CAAGGCAGGAGGTGTTTCTTTACTAGCAGC**
B3004	Reverse	5	AGTGTGTCATGCGTGT**CAAGGCAGGAGGTGTTTCTTTACTAGCAGC**
B3005	Reverse	6	GCATATAGTAGAGATC**CAAGGCAGGAGGTGTTTCTTTACTAGCAGC**
B3006	Reverse	7	CAGCAGTATAGACTGT**CAAGGCAGGAGGTGTTTCTTTACTAGCAGC**
B3007	Reverse	8	AGATGTAGCACATCAT**CAAGGCAGGAGGTGTTTCTTTACTAGCAGC**
B3008	Reverse	9	GTCGTAGAGCACGCAG**CAAGGCAGGAGGTGTTTCTTTACTAGCAGC**
B2745	Reverse	10	AGTCATCGTATCGCGC**CAAGGCAGGAGGTGTTTCTTTACTAGCAGC**
B2746	Reverse	11	CGATCAGCTGAGCGCG**CAAGGCAGGAGGTGTTTCTTTACTAGCAGC**
B2747	Reverse	12	TCTGTAGTGCGTGCGC**CAAGGCAGGAGGTGTTTCTTTACTAGCAGC**
B2748	Reverse	13	GTCGCGACGTCAGTGT**CAAGGCAGGAGGTGTTTCTTTACTAGCAGC**
B2749	Reverse	14	TATACGTATATAGACG**CAAGGCAGGAGGTGTTTCTTTACTAGCAGC**
B2750	Reverse	15	AGCTCTGAGTCTCTAT**CAAGGCAGGAGGTGTTTCTTTACTAGCAGC**
B2751	Reverse	16	TCTACTCTCGCATCTA**CAAGGCAGGAGGTGTTTCTTTACTAGCAGC**
B2752	Reverse	17	CACGATAGTCGCTATG**CAAGGCAGGAGGTGTTTCTTTACTAGCAGC**
B2753	Reverse	18	ATCTAGCGTAGTGATG**CAAGGCAGGAGGTGTTTCTTTACTAGCAGC**
B2762	Reverse	19	TGCATGCACAGATGCG**CAAGGCAGGAGGTGTTTCTTTACTAGCAGC**
B3009	Reverse	20	GAGAGACGATCACATA**CAAGGCAGGAGGTGTTTCTTTACTAGCAGC**
B3010	Reverse	21	TAGTCACAGCGTGTAC**CAAGGCAGGAGGTGTTTCTTTACTAGCAGC**
B3011	Reverse	22	AGATATGCGCGACACG**CAAGGCAGGAGGTGTTTCTTTACTAGCAGC**
B3012	Reverse	23	TATGCATGACTGATAT**CAAGGCAGGAGGTGTTTCTTTACTAGCAGC**
B3013	Reverse	24	CGAGACTGTCGATCTC**CAAGGCAGGAGGTGTTTCTTTACTAGCAGC**
B3014	Reverse	25	CGCGCGTGTGTGCGTG**CAAGGCAGGAGGTGTTTCTTTACTAGCAGC**
B3015	Reverse	26	CACACGCGCGTGCTCG**CAAGGCAGGAGGTGTTTCTTTACTAGCAGC**
B3016	Reverse	27	ATCTGTGCGAGACTAC**CAAGGCAGGAGGTGTTTCTTTACTAGCAGC**
B3017	Reverse	28	ACGCGCACAGAGTCTC**CAAGGCAGGAGGTGTTTCTTTACTAGCAGC**
B3018	Reverse	29	TCTCTCACAGTCGAGC**CAAGGCAGGAGGTGTTTCTTTACTAGCAGC**
B3019	Reverse	30	GTCATCACACATCTCT**CAAGGCAGGAGGTGTTTCTTTACTAGCAGC**
B3020	Reverse	31	CTGATATGTAGTCGTA**CAAGGCAGGAGGTGTTTCTTTACTAGCAGC**
B3021	Reverse	32	AGACTCTACAGAGATA**CAAGGCAGGAGGTGTTTCTTTACTAGCAGC**
B3022	Reverse	33	GCGCATGTCTCTCTCT**CAAGGCAGGAGGTGTTTCTTTACTAGCAGC**
B3023	Reverse	34	TCTACAGAGCGAGAGT**CAAGGCAGGAGGTGTTTCTTTACTAGCAGC**
B3024	Reverse	35	ACTACTGAGACATAGA**CAAGGCAGGAGGTGTTTCTTTACTAGCAGC**
B3025	Reverse	36	CGCATGAGATATACGC**CAAGGCAGGAGGTGTTTCTTTACTAGCAGC**
B3026	Reverse	37	GTAGCGACATACGCAC**CAAGGCAGGAGGTGTTTCTTTACTAGCAGC**
B3027	Reverse	38	TGTGATACTGCGAGCA**CAAGGCAGGAGGTGTTTCTTTACTAGCAGC**

Table S2: Samples and Barcoding

Shorthand	GCB Number	Gene Knockout	Infecting Strain (GCB)	Mouse	Weeks pi	Tissue	Barcode
WT.M1.H	1468	*WT*	933	1	5	Heart	F1-R1
WT.M1.B	1469	*WT*	933	1	5	Bladder	F1-R1
WT.M1.J	1470	*WT*	933	1	5	Joint	F1-R1
WT.M1.E	1471	*WT*	933	1	5	Ear	F1-R1
WT.M2.H	1472	*WT*	933	2	5	Heart	F2-R2
WT.M2.B	1473	*WT*	933	2	5	Bladder	F2-R2
WT.M2.J	1474	*WT*	933	2	5	Joint	F2-R2
WT.M2.E	1475	*WT*	933	2	5	Ear	F2-R2
bbg32.C6.M1.H	1476	*bbg32.C6*	1233	1	5	Heart	F3-R3
bbg32.C6.M1.B	1477	*bbg32.C6*	1233	1	5	Bladder	F3-R3
bbg32.C6.M1.J	1478	*bbg32.C6*	1233	1	5	Joint	F3-R3
bbg32.C6.M1.E	1479	*bbg32.C6*	1233	1	5	Ear	F3-R3
bbg32.C6.M2.H	1480	*bbg32.C6*	1233	2	5	Heart	F4-R4
bbg32.C6.M2.B	1481	*bbg32.C6*	1233	2	5	Bladder	F4-R4
bbg32.C6.M2.J	1482	*bbg32.C6*	1233	2	5	Joint	F4-R4
bbg32.C6.M2.E	1483	*bbg32.C6*	1233	2	5	Ear	F4-R4
bbg32.C7.M1.H	1484	*bbg32.C7*	1234	1	5	Heart	F5-R5
bbg32.C7.M1.B	1485	*bbg32.C7*	1234	1	5	Bladder	F5-R5
bbg32.C7.M1.J	1486	*bbg32.C7*	1234	1	5	Joint	F5-R5
bbg32.C7.M1.E	1487	*bbg32.C7*	1234	1	5	Ear	F5-R5
bbg32.C7.M2.H	1488	*bbg32.C7*	1234	2	5	Heart	F6-R6
bbg32.C7.M2.B	1489	*bbg32.C7*	1234	2	5	Bladder	F6-R6
bbg32.C7.M2.J	1490	*bbg32.C7*	1234	2	5	Joint	F6-R6
bbg32.C7.M2.E	1491	*bbg32.C7*	1234	2	5	Ear	F6-R6
sbcC.C2.M1.H	1492	*sbcC.C2*	1248	1	5	Heart	F7-R7
sbcC.C2.M1.B	1493	*sbcC.C2*	1248	1	5	Bladder	F7-R7
sbcC.C2.M1.J	1494	*sbcC.C2*	1248	1	5	Joint	F7-R7
sbcC.C2.M1.E	1495	*sbcC.C2*	1248	1	5	Ear	F7-R7
sbcC.C2.M2.H	1496	*sbcC.C2*	1248	2	5	Heart	F8-R8
sbcC.C2.M2.B	1497	*sbcC.C2*	1248	2	5	Bladder	F8-R8
sbcC.C2.M2.J	1498	*sbcC.C2*	1248	2	5	Joint	F8-R8
sbcC.C2.M2.E	1499	*sbcC.C2*	1248	2	5	Ear	F8-R8
sbcC.C3.M1.H	1600	*sbcC.C3*	1249	1	5	Heart	F9-R9
sbcC.C3.M1.B	1601	*sbcC.C3*	1249	1	5	Bladder	F9-R9
sbcC.C3.M1.J	1602	*sbcC.C3*	1249	1	5	Joint	F9-R9
sbcC.C3.M1.E	1603	*sbcC.C3*	1249	1	5	Ear	F9-R9
sbcC.C3.M2.H	1604	*sbcC.C3*	1249	2	5	Heart	F10-R10
sbcC.C3.M2.B	1605	*sbcC.C3*	1249	2	5	Bladder	F10-R10

sbcC.C3.M2.J	1606	*sbcC.C3*	1249	2	5	Joint	F10-R10
sbcC.C3.M2.E	1607	*sbcC.C3*	1249	2	5	Ear	F10-R10
sbcD.C1.M1.H	1608	*sbcD.C1*	1251	1	5	Heart	F11-R11
sbcD.C1.M1.B	1609	*sbcD.C1*	1251	1	5	Bladder	F11-R11
sbcD.C1.M1.J	1610	*sbcD.C1*	1251	1	5	Joint	F11-R11
sbcD.C1.M1.E	1611	*sbcD.C1*	1251	1	5	Ear	F11-R11
sbcD.C1.M2.H	1612	*sbcD.C1*	1251	2	5	Heart	F12-R12
sbcD.C1.M2.B	1613	*sbcD.C1*	1251	2	5	Bladder	F12-R12
sbcD.C1.M2.J	1614	*sbcD.C1*	1251	2	5	Joint	F12-R12
sbcD.C1.M2.E	1615	*sbcD.C1*	1251	2	5	Ear	F12-R12
sbcD.C2.M1.H	1616	*sbcD.C2*	1252	1	5	Heart	F13-R13
sbcD.C2.M1.B	1617	*sbcD.C2*	1252	1	5	Bladder	F13-R13
sbcD.C2.M1.J	1618	*sbcD.C2*	1252	1	5	Joint	F13-R13
sbcD.C2.M1.E	1619	*sbcD.C2*	1252	1	5	Ear	F13-R13
sbcD.C2.M2.H	1620	*sbcD.C2*	1252	2	5	Heart	F14-R14
sbcD.C2.M2.B	1621	*sbcD.C2*	1252	2	5	Bladder	F14-R14
WT.M3.H	1622	*WT*	933	3	5	Heart	F15-R15
WT.M3.B	1623	*WT*	933	3	5	Bladder	F15-R15
WT.M3.J	1624	*WT*	933	3	5	Joint	F15-R15
WT.M3.E	1625	*WT*	933	3	5	Ear	F15-R15
WT.M4.H	1626	*WT*	933	4	5	Heart	F16-R16
WT.M4.B	1627	*WT*	933	4	5	Bladder	F16-R16
WT.M4.J	1628	*WT*	933	4	5	Joint	F16-R16
WT.M4.E	1629	*WT*	933	4	5	Ear	F16-R16
priA.C3.M1.H	1633	*priA.C3*	1206	1	5	Heart	F17-R17
priA.C3.M1.B	1634	*priA.C3*	1206	1	5	Bladder	F17-R17
priA.C3.M1.J	1635	*priA.C3*	1206	1	5	Joint	F17-R17
priA.C3.M1.E	1636	*priA.C3*	1206	1	5	Ear	F17-R17
priA.C3.M2.H	1637	*priA.C3*	1206	2	5	Heart	F18-R18
priA.C3.M2.B	1638	*priA.C3*	1206	2	5	Bladder	F18-R18
priA.C3.M2.J	1639	*priA.C3*	1206	2	5	Joint	F18-R18
priA.C3.M2.E	1640	*priA.C3*	1206	2	5	Ear	F18-R18
recJ.C1.M1.H	1641	*recJ.C1*	1153	1	5	Heart	F19-R19
recJ.C1.M1.B	1642	*recJ.C1*	1153	1	5	Bladder	F19-R19
recJ.C1.M1.J	1643	*recJ.C1*	1153	1	5	Joint	F19-R19
recJ.C1.M1.E	1644	*recJ.C1*	1153	1	5	Ear	F19-R19
recJ.C1.M2.H	1645	*recJ.C1*	1153	2	5	Heart	F20-R20
recJ.C1.M2.B	1646	*recJ.C1*	1153	2	5	Bladder	F20-R20
recJ.C1.M2.J	1647	*recJ.C1*	1153	2	5	Joint	F20-R20
recJ.C1.M2.E	1648	*recJ.C1*	1153	2	5	Ear	F20-R20
recJ.C5.M1.H	1649	*recJ.C5*	1154	1	5	Heart	F21-R21
recJ.C5.M1.B	1650	*recJ.C5*	1154	1	5	Bladder	F21-R21

recJ.C5.M1.J	1651	*recJ.C5*	1154	1	5	Joint	F21-R21
recJ.C5.M1.E	1652	*recJ.C5*	1154	1	5	Ear	F21-R21
recJ.C5.M2.H	1653	*recJ.C5*	1154	2	5	Heart	F22-R22
recJ.C5.M2.B	1654	*recJ.C5*	1154	2	5	Bladder	F22-R22
recJ.C5.M2.J	1655	*recJ.C5*	1154	2	5	Joint	F22-R22
recJ.C5.M2.E	1656	*recJ.C5*	1154	2	5	Ear	F22-R22
mutL.C1.M1.H	1657	*mutL.C1*	1178	1	5	Heart	F23-R23
mutL.C1.M1.B	1658	*mutL.C1*	1178	1	5	Bladder	F23-R23
mutL.C1.M1.J	1659	*mutL.C1*	1178	1	5	Joint	F23-R23
mutL.C1.M1.E	1660	*mutL.C1*	1178	1	5	Ear	F23-R23
mutL.C1.M2.H	1661	*mutL.C1*	1178	2	5	Heart	F24-R24
mutL.C1.M2.B	1662	*mutL.C1*	1178	2	5	Bladder	F24-R24
mutL.C1.M2.J	1663	*mutL.C1*	1178	2	5	Joint	F24-R24
mutL.C1.M2.E	1664	*mutL.C1*	1178	2	5	Ear	F24-R24
mutL.C2.M1.H	1665	*mutL.C2*	1179	1	5	Heart	F25-R25
mutL.C2.M1.B	1666	*mutL.C2*	1179	1	5	Bladder	F25-R25
mutL.C2.M1.J	1667	*mutL.C2*	1179	1	5	Joint	F25-R25
mutL.C2.M1.E	1668	*mutL.C2*	1179	1	5	Ear	F25-R25
mutL.C2.M2.H	1669	*mutL.C2*	1179	2	5	Heart	F26-R26
mutL.C2.M2.B	1670	*mutL.C2*	1179	2	5	Bladder	F26-R26
mutL.C2.M2.J	1671	*mutL.C2*	1179	2	5	Joint	F26-R26
mutL.C2.M2.E	1672	*mutL.C2*	1179	2	5	Ear	F26-R26
WT.W0	933	*WT*	933	NA	0	NA	F27-R27
bbg32.C6.W0	1233	*bbg32.C6*	1233	NA	0	NA	F28-R28
bbg32.C7.W0	1234	*bbg32.C7*	1234	NA	0	NA	F29-R29
sbcC.C2.W0	1248	*sbcC.C2*	1248	NA	0	NA	F30-R30
sbcC.C3.W0	1249	*sbcC.C3*	1249	NA	0	NA	F31-R31
sbcD.C1.W0	1251	*sbcD.C1*	1251	NA	0	NA	F32-R32
sbcD.C2.W0	1252	*sbcD.C2*	1252	NA	0	NA	F33-R33
priA.C3.W0	1206	*priA.C3*	1206	NA	0	NA	F34-R34
recJ.C1.W0	1153	*recJ.C1*	1153	NA	0	NA	F35-R35
recJ.C5.W0	1154	*recJ.C5*	1154	NA	0	NA	F36-R36
mutL.C1.W0	1178	*mutL.C1*	1178	NA	0	NA	F37-R37
mutL.C2.W0	1179	*mutL.C2*	1179	NA	0	NA	F38-R38
WT.C2.M1.W1	4608	*WT.C2*	2958	1	1	Blood	F1-R10
WT.C2.M2.W1	4609	*WT.C2*	2958	2	1	Blood	F2-R10
WT.C3.M1.W1	4610	*WT.C3*	2959	1	1	Blood	F3-R10
WT.C3.M2.W1	4611	*WT.C3*	2959	2	1	Blood	F4-R10
mutL.C1.M1.W1	4612	*mutL.C1*	1178	1	1	Blood	F5-R10
mutL.C1.M2.W1	4613	*mutL.C1*	1178	2	1	Blood	F6-R10

mutL.C2.M1.W1	4614	*mutL.C2*	1179	1	1	Blood	F7-R10
mutL.C2.M2.W1	4615	*mutL.C2*	1179	2	1	Blood	F8-R10
Bind.C6.M1.W1	4616	*mutL* ATP binding C6	4529	1	1	Blood	F9-R10
Bind.C6.M2.W1	4617	*mutL* ATP binding C6	4529	2	1	Blood	F10-R10
Bind.C10.M1.W1	4618	*mutL* ATP binding C10	4530	1	1	Blood	F11-R10
Bind.C10.M2.W1	4619	*mutL* ATP binding C10	4530	2	1	Blood	F12-R10
Endo.C6.M1.W1	4620	*mutL* endo C6	4520	1	1	Blood	F1-R11
Endo.C6.M2.W1	4621	*mutL* endo C6	4520	2	1	Blood	F2-R11
Endo.C12.M1.W1	4622	*mutL* endo C12	4521	1	1	Blood	F3-R11
Endo.C12.M2.W1	4623	*mutL* endo C12	4521	2	1	Blood	F4-R11
Hydro.C15.M1.W1	4626	*mutL* ATP hydrolysis C15	4528	1	1	Blood	F7-R11
Hydro.C15.M2.W1	4627	*mutL* ATP hydrolysis C15	4528	2	1	Blood	F8-R11
Clamp.C6.M1.W1	4676	*mutL* β-clamp binding C6	4533	1	1	Blood	F9-R11
Clamp.C6.M2.W1	4629	*mutL* β-clamp binding C6	4533	2	1	Blood	F10-R11
Clamp.C8.M1.W1	4630	*mutL* β-clamp binding C8	4534	1	1	Blood	F11-R11
Clamp.C8.M2.W1	4678	*mutL* β-clamp binding C8	4534	2	1	Blood	F12-R11
WT.C2.M1.W2	4632	*WT.C2*	2958	1	2	Ear	F1-R12
WT.C2.M2.W2	4633	*WT.C2*	2958	2	2	Ear	F2-R12
WT.C3.M1.W2	4634	*WT.C3*	2959	1	2	Ear	F3-R12
WT.C3.M2.W2	4635	*WT.C3*	2959	2	2	Ear	F4-R12
mutL.C1.M1.W2	4636	*mutL.C1*	1178	1	2	Ear	F5-R12
mutL.C1.M2.W2	4637	*mutL.C1*	1178	2	2	Ear	F6-R12
mutL.C2.M1.W2	4638	*mutL.C2*	1179	1	2	Ear	F7-R12
mutL.C2.M2.W2	4639	*mutL.C2*	1179	2	2	Ear	F8-R12
Bind.C6.M1.W2	C*	*mutL* ATP binding C6	4529	1	2	Ear	NA
Bind.C6.M2.W2	4641	*mutL* ATP binding C6	4529	2	2	Ear	F10-R12
Bind.C10.M1.W2	4642	*mutL* ATP binding C10	4530	1	2	Ear	F11-R12

Bind.C10.M2.W2	4643	*mutL* ATP binding C10	4530	2	2	Ear	F12-R12
Endo.C6.M1.W2	4644	*mutL* endo C6	4520	1	2	Ear	F1-R13
Endo.C6.M2.W2	4645	*mutL* endo C6	4520	2	2	Ear	F2-R13
Endo.C12.M1.W2	4646	*mutL* endo C12	4521	1	2	Ear	F3-R13
Endo.C12.M2.W2	4647	*mutL* endo C12	4521	2	2	Ear	F4-R13
Hydro.C15.M1.W2	4650	*mutL* ATP hydrolysis C15	4528	1	2	Ear	F7-R13
Hydro.C15.M2.W2	4651	*mutL* ATP hydrolysis C15	4528	2	2	Ear	F8-R13
Clamp.C6.M1.W2	4723	*mutL* β-clamp binding C6	4533	1	2	Ear	F9-R13
Clamp.C6.M2.W2	4652	*mutL* β-clamp binding C6	4533	2	2	Ear	F10-R13
Clamp.C8.M1.W2	4653	*mutL* β-clamp binding C8	4534	1	2	Ear	F11-R13
Clamp.C8.M2.W2	4724	*mutL* β-clamp binding C8	4534	2	2	Ear	F12-R13
WT.C2.M1.W3	4654	*WT.C2*	2958	1	3	Ear	F1-R14
WT.C2.M2.W3	4655	*WT.C2*	2958	2	3	Ear	F2-R14
WT.C3.M1.W3	4656	*WT.C3*	2959	1	3	Ear	F3-R14
WT.C3.M2.W3	4657	*WT.C3*	2959	2	3	Ear	F4-R14
mutL.C1.M1.W3	4658	*mutL.C1*	1178	1	3	Ear	F5-R14
mutL.C1.M2.W3	4659	*mutL.C1*	1178	2	3	Ear	F6-R14
mutL.C2.M1.W3	4660	*mutL.C2*	1179	1	3	Ear	F7-R14
mutL.C2.M2.W3	4661	*mutL.C2*	1179	2	3	Ear	F8-R14
Bind.C6.M1.W3	4662	*mutL* ATP binding C6	4529	1	3	Ear	F9-R14
Bind.C6.M2.W3	4663	*mutL* ATP binding C6	4529	2	3	Ear	F10-R14
Bind.C10.M1.W3	4664	*mutL* ATP binding C10	4530	1	3	Ear	F11-R14
Bind.C10.M2.W3	4665	*mutL* ATP binding C10	4530	2	3	Ear	F12-R14
Endo.C6.M1.W3	4666	*mutL* endo C6	4520	1	3	Ear	F1-R15
Endo.C6.M2.W3	4667	*mutL* endoC6	4520	2	3	Ear	F2-R15
Endo.C12.M1.W3	4668	*mutL* endo C12	4521	1	3	Ear	F3-R15
Endo.C12.M2.W3	4669	*mutL* endo C12	4521	2	3	Ear	F4-R15

Hydro.C15.M1.W3	4672	*mutL* ATP hydrolysis C15	4528	1	3	Ear	F7-R15
Hydro.C15.M2.W3	4673	*mutL* ATP hydrolysis C15	4528	2	3	Ear	F8-R15
Clamp.C6.M1.W3	4725	*mutL* β-clamp binding C6	4533	1	3	Ear	F9-R15
Clamp.C6.M2.W3	4674	*mutL* β-clamp binding C6	4533	2	3	Ear	F10-R15
Clamp.C8.M1.W3	4675	*mutL* β-clamp binding C8	4534	1	3	Ear	F11-R15
Clamp.C8.M2.W3	4726	*mutL* β-clamp binding C8	4534	2	3	Ear	F12-R15
WT.C2.M1.W4.E	4679	*WT.C2*	2958	1	4	Ear	F1-R16
WT.C2.M2.W4.E	4681	*WT.C2*	2958	2	4	Ear	F2-R16
WT.C3.M1.W4.E	4683	*WT.C3*	2959	1	4	Ear	F3-R16
WT.C3.M2.W4.E	4685	*WT.C3*	2959	2	4	Ear	F4-R16
mutL.C1.M1.W4.E	4687	*mutL.C1*	1178	1	4	Ear	F5-R16
mutL.C1.M2.W4.E	4689	*mutL.C1*	1178	2	4	Ear	F6-R16
mutL.C2.M1.W4.E	4691	*mutL.C2*	1179	1	4	Ear	F7-R16
mutL.C2.M2.W4.E	4693	*mutL.C2*	1179	2	4	Ear	F8-R16
Bind.C6.M1.W4.E	5695	*mutL* ATP binding C6	4529	1	4	Ear	F9-R16
Bind.C6.M2.W4.E	4697	*mutL* ATP binding C6	4529	2	4	Ear	F10-R16
Bind.C10.M1.W4.E	4699	*mutL* ATP binding C10	4530	1	4	Ear	F11-R16
Bind.C10.M2.W4.E	4701	*mutL* ATP binding C10	4530	2	4	Ear	F12-R16
Endo.C6.M1.W4.E	4703	*mutL* endo C6	4520	1	4	Ear	F1-R17
Endo.C6.M2.W4.E	4705	*mutL* endo C6	4520	2	4	Ear	F2-R17
Endo.C12.M1.W4.E	4707	*mutL* endo C12	4521	1	4	Ear	F3-R17
Endo.C12.M2.W4.E	4709	*mutL* endo C12	4521	2	4	Ear	F4-R17
Hydro.C15.M1.W4.E	4715	*mutL* ATP hydrolysis C15	4528	1	4	Ear	F7-R17
Hydro.C15.M2.W4.E	4717	*mutL* ATP hydrolysis C15	4528	2	4	Ear	F8-R17
Clamp.C6.M1.W4.E	4727	*mutL* β-clamp binding C6	4533	1	4	Ear	F9-R17
Clamp.C6.M2.W4.E	4719	*mutL* β-clamp binding C6	4533	2	4	Ear	F10-R17
Clamp.C8.M1.W4.E	4721	*mutL* β-clamp binding C8	4534	1	4	Ear	F11-R17

Clamp.C8.M2.W4.E	4729	*mutL* β-clamp binding C8	4534	2	4	Ear	F12-R17
WT.C2.M1.W4.B	4680	*WT.C2*	2958	1	4	Bladder	F1-R18
WT.C2.M2.W4.B	4682	*WT.C2*	2958	2	4	Bladder	F2-R18
WT.C3.M1.W4.B	4684	*WT.C3*	2959	1	4	Bladder	F3-R18
WT.C3.M2.W4.B	4686	*WT.C3*	2959	2	4	Bladder	F4-R18
mutL.C1.M1.W4.B	4688	*mutL.C1*	1178	1	4	Bladder	F5-R18
mutL.C1.M2.W4.B	4690	*mutL.C1*	1178	2	4	Bladder	F6-R18
mutL.C2.M1.W4.B	4692	*mutL.C2*	1179	1	4	Bladder	F7-R18
mutL.C2.M2.W4.B	4694	*mutL.C2*	1179	2	4	Bladder	F8-R18
Bind.C6.M1.W4.B	C*	*mutL* ATP binding C6	4529	1	4	Bladder	NA
Bind.C6.M2.W4.B	4698	*mutL* ATP binding C6	4529	2	4	Bladder	F10-R18
Bind.C10.M1.W4.B	4700	*mutL* ATP binding C10	4530	1	4	Bladder	F11-R18
Bind.C10.M2.W4.B	4702	*mutL* ATP binding C10	4530	2	4	Bladder	F12-R18
Endo.C6.M1.W4.B	4704	*mutL* endo C6	4520	1	4	Bladder	F1-R19
Endo.C6.M2.W4.B	4706	*mutL* endo C6	4520	2	4	Bladder	F2-R19
Endo.C12.M1.W4.B	4708	*mutL* endo C12	4521	1	4	Bladder	F3-R19
Endo.C12.M2.W4.B	4710	*mutL* endo C12	4521	2	4	Bladder	F4-R19
Hydro.C15.M1.W4.B	4716	*mutL* ATP hydrolysis C15	4528	1	4	Bladder	F7-R19
Hydro.C15.M2.W4.B	4718	*mutL* ATP hydrolysis C15	4528	2	4	Bladder	F8-R19
Clamp.C6.M1.W4.B	4728	*mutL* β-clamp binding C6	4533	1	4	Bladder	F9-R19
Clamp.C6.M2.W4.B	4720	*mutL* β-clamp binding C6	4533	2	4	Bladder	F10-R19
Clamp.C8.M1.W4.B	4722	*mutL* β-clamp binding C8	4534	1	4	Bladder	F11-R19
Clamp.C8.M2.W4.B	4730	*mutL* β-clamp binding C8	4534	2	4	Bladder	F12-R19

NA = not applicable; C* = contaminated sample, could not be recovered; Black bar separates the sequencing of mutants described earlier (Dresser et al., 2009) from those constructed in this study.

Table S3. Strains and plasmids used in this study

Strain number	Strain	Plasmid /or KO Plasmid	Drug	Description or mutation	Reference
GCE719	DH5-alpha			*E. coli* strain used for cloning	(Taylor et al., 1993)
GCE32	DH5-alpha	pOK12	Kan	Backbone	(Vieira and Messing, 1991)
GCE3829	DH5-alpha	pMC120	Carb	pJET::*mutL* (ATP binding)	This study
GCE3831	DH5-alpha	pMC121	Carb	pJET::*mutL* (β-clamp binding)	"
GCE3833	DH5-alpha	pMC122	Carb	pJET::*mutL* (endonuclease)	"
GCE3835	DH5-alpha	pMC123	Carb	pJET::*mutL* (ATP hydrolysis)	"
GCE3868	DH5-alpha	pMC140	Kan	pOK12::*mutL* (endonuclease)	"
GCE3871	DH5-alpha	pMC141	Kan	pOK12::*mutL* (ATP binding)	"
GCE3874	DH5-alpha	pMC142	Kan	pOK12::*mutL* (β-clamp binding)	"
GCE3877	DH5-alpha	pMC143	Kan	pOK12::*mutL* (ATP hydrolysis)	"
GCE3880	DH5-alpha	pMC144	Kan/Gent	pOK12::*mutL* (ATP binding)::Gent	"
GCE3882	DH5-alpha	pMC145	Kan/Gent	pOK12::*mutL* (β-clamp binding)::Gent	"
GCE3884	DH5-alpha	pMC146	Kan/Gent	pOK12::*mutL* ATP hydrolysis)::Gent	"
GCE3886	DH5-alpha	pMC147	Kan/Gent	pOK12::*mutL* (endonuclease)::Gent	"
GCE3888	DH5-alpha	pMC148	Kan/Gent	pOK12::*mutL* (ATP binding)::Gent::bb*0212*	"
GCE3890	DH5-alpha	pMC149	Kan/Gent	pOK12::*mutL* (ATP hydrolysis)::Gent::*bb0212*	"
GCE3892	DH5-alpha	pMC150	Kan/Gent	pOK12::*mutL* (endonuclease)::Gent::*bb0212*	"
GEC3894	DH5-alpha	pMC151	Kan/Gent	pOK12::*mutL* (β-clamp binding)::Gent::*bb0212*	"
GCB933	5A4			Wild type	(Purser and Norris, 2000)
GCB1153	5A4	pAD51	Gent	*recJ* Clone 1	(Dresser et al., 2009)
GCB1154	5A4	pAD51	Gent	*recJ* Clone 5	"
GCB1178	5A4	pAD61	Gent	*mutL* Clone 1	"
GCB1179	5A4	pAD61	Gent	*mutL* Clone 2	"
GCB1206	5A4	pAD96	Gent	*priA* Clone 3	"
GCB1233	5A4	pAD88	Gent	*bbg32* Clone 6	"
GCB1234	5A4	pAD88	Gent	*bbg32* Clone 7	"
GCB1248	5A4	pAD97	Gent	*sbcC* Clone 2	"
GCB1249	5A4	pAD97	Gent	*sbcC* Clone 3	"
GCB1251	5A4	pAD87	Gent	*sbcD* Clone 1	"
GCB1252	5A4	pAD87	Gent	*sbcD* Clone 2	"
GCB1468	5A4		Gent	*WT*.M1.H	"
GCB1469	5A4		Gent	*WT*.M1.B	"
GCB1470	5A4		Gent	*WT*.M1.J	"
GCB1471	5A4		Gent	*WT*.M1.E	"
GCB1472	5A4		Gent	*WT*.M2.H	"
GCB1473	5A4		Gent	*WT*.M2.B	"
GCB1474	5A4		Gent	*WT*.M2.J	"
GCB1475	5A4		Gent	*WT*.M2.E	"

GCB1476	5A4	pAD88	Gent	*bbg32*.C6.M1.H	"
GCB1477	5A4	pAD88	Gent	*bbg32*.C6.M1.B	"
GCB1478	5A4	pAD88	Gent	*bbg32*.C6.M1.J	"
GCB1479	5A4	pAD88	Gent	*bbg32*.C6.M1.E	"
GCB1480	5A4	pAD88	Gent	*bbg32*.C6.M2.H	"
GCB1481	5A4	pAD88	Gent	*bbg32*.C6.M2.B	"
GCB1482	5A4	pAD88	Gent	*bbg32*.C6.M2.J	"
GCB1483	5A4	pAD88	Gent	*bbg32*.C6.M2.E	"
GCB1484	5A4	pAD88	Gent	*bbg32*.C7.M1.H	"
GCB1485	5A4	pAD88	Gent	*bbg32*.C7.M1.B	"
GCB1486	5A4	pAD88	Gent	*bbg32*.C7.M1.J	"
GCB1487	5A4	pAD88	Gent	*bbg32*.C7.M1.E	"
GCB1488	5A4	pAD88	Gent	*bbg32*.C7.M2.H	"
GCB1489	5A4	pAD88	Gent	*bbg32*.C7.M2.B	"
GCB1490	5A4	pAD88	Gent	*bbg32*.C7.M2.J	"
GCB1491	5A4	pAD88	Gent	*bbg32*.C7.M2.E	"
GCB1492	5A4	pAD97	Gent	*sbcC*.C2.M1.H	"
GCB1493	5A4	pAD97	Gent	*sbcC*.C2.M1.B	"
GCB1494	5A4	pAD97	Gent	*sbcC*.C2.M1.J	"
GCB1495	5A4	pAD97	Gent	*sbcC*.C2.M1.E	"
GCB1496	5A4	pAD97	Gent	*sbcC*.C2.M2.H	"
GCB1497	5A4	pAD97	Gent	*sbcC*.C2.M2.B	"
GCB1498	5A4	pAD97	Gent	*sbcC*.C2.M2.J	"
GCB1499	5A4	pAD97	Gent	*sbcC*.C2.M2.E	"
GCB1600	5A4	pAD97	Gent	*sbcC*.C3.M1.H	"
GCB1601	5A4	pAD97	Gent	*sbcC*.C3.M1.B	"
GCB1602	5A4	pAD97	Gent	*sbcC*.C3.M1.J	"
GCB1603	5A4	pAD97	Gent	*sbcC*.C3.M1.E	"
GCB1604	5A4	pAD97	Gent	*sbcC*.C3.M2.H	"
GCB1605	5A4	pAD97	Gent	*sbcC*.C3.M2.B	"
GCB1606	5A4	pAD97	Gent	*sbcC*.C3.M2.J	"
GCB1607	5A4	pAD97	Gent	*sbcC*.C3.M2.E	"
GCB1608	5A4	pAD87	Gent	*sbcD*.C1.M1.H	"
GCB1609	5A4	pAD87	Gent	*sbcD*.C1.M1.B	"
GCB1610	5A4	pAD87	Gent	*sbcD*.C1.M1.J	"
GCB1611	5A4	pAD87	Gent	*sbcD*.C1.M1.E	"
GCB1612	5A4	pAD87	Gent	*sbcD*.C1.M2.H	"
GCB1613	5A4	pAD87	Gent	*sbcD*.C1.M2.B	"
GCB1614	5A4	pAD87	Gent	*sbcD*.C1.M2.J	"
GCB1615	5A4	pAD87	Gent	*sbcD*.C1.M2.E	"
GCB1616	5A4	pAD87	Gent	*sbcD*.C2.M1.H	"
GCB1617	5A4	pAD87	Gent	*sbcD*.C2.M1.B	"
GCB1618	5A4	pAD87	Gent	*sbcD*.C2.M1.J	"
GCB1619	5A4	pAD87	Gent	*sbcD*.C2.M1.E	"
GCB1620	5A4	pAD87	Gent	*sbcD*.C2.M2.H	"
GCB1621	5A4	pAD87	Gent	*sbcD*.C2.M2.B	"
GCB1622	5A4		Gent	*WT*.M3.H	"
GCB1623	5A4		Gent	*WT*.M3.B	"
GCB1624	5A4		Gent	*WT*.M3.J	"
GCB1625	5A4		Gent	*WT*.M3.E	"

GCB1626	5A4		Gent	*WT*.M4.H	"
GCB1627	5A4		Gent	*WT*.M4.B	"
GCB1628	5A4		Gent	*WT*.M4.J	"
GCB1629	5A4		Gent	*WT*.M4.E	"
GCB1633	5A4	pAD96	Gent	*priA*.C3.M1.H	"
GCB1634	5A4	pAD96	Gent	*priA*.C3.M1.B	"
GCB1635	5A4	pAD96	Gent	*priA*.C3.M1.J	"
GCB1636	5A4	pAD96	Gent	*priA*.C3.M1.E	"
GCB1637	5A4	pAD96	Gent	*priA*.C3.M2.H	"
GCB1638	5A4	pAD96	Gent	*priA*.C3.M2.B	"
GCB1639	5A4	pAD96	Gent	*priA*.C3.M2.J	"
GCB1640	5A4	pAD96	Gent	*priA*.C3.M2.E	"
GCB1641	5A4	pAD51	Gent	*recJ*.C1.M1.H	"
GCB1642	5A4	pAD51	Gent	*recJ*.C1.M1.B	"
GCB1643	5A4	pAD51	Gent	*recJ*.C1.M1.J	"
GCB1644	5A4	pAD51	Gent	*recJ*.C1.M1.E	"
GCB1645	5A4	pAD51	Gent	*recJ*.C1.M2.H	"
GCB1646	5A4	pAD51	Gent	*recJ*.C1.M2.B	"
GCB1647	5A4	pAD51	Gent	*recJ*.C1.M2.J	"
GCB1648	5A4	pAD51	Gent	*recJ*.C1.M2.E	"
GCB1649	5A4	pAD51	Gent	*recJ*.C5.M1.H	"
GCB1650	5A4	pAD51	Gent	*recJ*.C5.M1.B	"
GCB1651	5A4	pAD51	Gent	*recJ*.C5.M1.J	"
GCB1652	5A4	pAD51	Gent	*recJ*.C5.M1.E	"
GCB1653	5A4	pAD51	Gent	*recJ*.C5.M2.H	"
GCB1654	5A4	pAD51	Gent	*recJ*.C5.M2.B	"
GCB1655	5A4	pAD51	Gent	*recJ*.C5.M2.J	"
GCB1656	5A4	pAD51	Gent	*recJ*.C5.M2.E	"
GCB1657	5A4	pAD61	Gent	*mutL*.C1.M1.H	"
GCB1658	5A4	pAD61	Gent	*mutL*.C1.M1.B	"
GCB1659	5A4	pAD61	Gent	*mutL*.C1.M1.J	"
GCB1660	5A4	pAD61	Gent	*mutL*.C1.M1.E	"
GCB1661	5A4	pAD61	Gent	*mutL*.C1.M2.H	"
GCB1662	5A4	pAD61	Gent	*mutL*.C1.M2.B	"
GCB1663	5A4	pAD61	Gent	*mutL*.C1.M2.J	"
GCB1664	5A4	pAD61	Gent	*mutL*.C1.M2.E	"
GCB1665	5A4	pAD61	Gent	*mutL*.C2.M1.H	"
GCB1666	5A4	pAD61	Gent	*mutL*.C2.M1.B	"
GCB1667	5A4	pAD61	Gent	*mutL*.C2.M1.J	"
GCB1668	5A4	pAD61	Gent	*mutL*.C2.M1.E	"
GCB1669	5A4	pAD61	Gent	*mutL*.C2.M2.H	"
GCB1670	5A4	pAD61	Gent	*mutL*.C2.M2.B	"
GCB1671	5A4	pAD61	Gent	*mutL*.C2.M2.J	"
GCB1672	5A4	pAD61	Gent	*mutL*.C2.M2.E	"
GCB2958	5A4			Wild type (individual clone 2)	(Verhey et al., 2018)
GCB2959	5A4			Wild type (individual clone 3)	"
GCB4520	5A4	pMC150	Gent	*mutL* endonuclease mutant Clone 6	This study
GCB4521	5A4	pMC150	Gent	*mutL* endonuclease mutant Clone 12	This study
GCB4524	5A4	pMC149	Gent	*mutL* ATP hydrolysis mutant Clone 3	This study

GCB4525	5A4	pMC149	Gent	*mutL* ATP hydrolysis mutant Clone 4	This study
GCB4528	5A4	pMC149	Gent	*mutL* ATP hydrolysis mutant Clone 15	This study
GCB4529	5A4	pMC148	Gent	*mutL* ATP binding site mutant Clone 6	This study
GCB4530	5A4	pMC148	Gent	*mutL* ATP binding site mutant Clone 10	This study
GCB4533	5A4	pMC151	Gent	*mutL* β-clamp binding mutant Clone 6	This study
GCB4534	5A4	pMC151	Gent	*mutL* β-clamp binding mutant Clone 8	This study

Table S4. Primers used to build *mutL* point mutant constructs.

Oligo name	Sequence	Rest. site	To amplify or sequence
B3049	ctcgagtttttcagcaagatacaaagccatagatttaaatccaga		Fw oligo for *mutL* left half (amplifies part of the gene upstream of *mutL*)
B3050	ctcctactgctattttttgaaccaag		Rev oligo for *mutL* left half. Includes the ATP binding site point mutations that overlap with the right *mutL* half
B3051	ggttcaaaaaatagcagtaggagaatcaatagacaggccatgttc		Fw oligo for *mutL* right half. Includes the ATP binding site point mutations that overlap with the left *mutL* half
B3052	tgtaggagatcttctagaaagatttatgctctgtcaacttttttttcaag		Rev oligo for *mutL* right half. Used to sequence part of *mutL*
B3053	gaatggtctagtagttcccttaatattg		Rev oligo for *mutL* left half. Includes the ATP hydrolysis mutations that overlap with the right *mutL* half
B3054	ttaagggaactactagaccattcaatagattctggagctactaaaattgag		Fw oligo for *mutL* right half. Includes the ATP hydrolysis site mutations that overlap with the left *mutL* half
B3055	ctcgagtttttcagcaagatctctaggctttaggggagag		Fw oligo for *mutL* left half
B3056	gtgttggtttatgaagtaaatttcatttatttttcaacg		Rev oligo for *mutL* left half. Includes the endonuclease site mutation that overlaps with the right *mutL* half
B3057	aaatgaaatttacttcataaaccaacacgcagttcac		Fw oligo for *mutL* right half. Includes the endonuclease site mutation that overlaps with the left *mutL* half
B3058	ttggtgctgcaagttttgcaacatttttttttgaatttctaagtttttcat		Rev oligo for *mutL* left half. Includes the β-clamp binding site mutations that overlap with the right *mutL* half
B3059	ttgcaaaacttgcagcaccaattgaattcacagtagttga		Fw oligo for *mutL* right half. Includes the β-clamp binding site mutations that overlap with the left *mutL* half
B3061	ttt*ccatgg*ttaggtggcggtacttgg	NcoI	Gent
B3094	ttt*ccatgg*tgcctggcagtaagttga	NcoI	Gent
B3101	tga*gagctc*agttgacagagcataaaataaaa	SacI	*bb0212* (gene downstream of *mutL*)
B3102	gct*ggatcc*attggttttggctgtca	Bam HI	*bb0212* (gene downstream of *mutL*) For analysis of merodiploidy in the ATP binding, hydrolysis or endonuclease site mutants, pairs with B3110
B3107	cttttccttgctgggaagttgt		To sequence *mutL*. Pairs with B3113 and B3114 for point mutation screening in *Bb*. Pairs with B3122 and amplifies a *mutL* stretch including point mutations for either the ATP binding or hydrolysis site
B3108	acccgccataaacggaaca		To sequence *mutL*
B3109	ctttctcctggcaacttccc		To sequence *mutL*. Pairs with B3124 and amplifies a *mutL* stretch including point mutations for either the endonuclease or the β-clamp binding

			site
B3110	ttagaatttgaagaacctccaaa		To sequence *mutL*. For analysis of merodiploidy, pairs with B3102 or B3128
B3113	tggttcaaaaaatagcagt		To screen for the introduction of the ATP binding site point mutations
B3114	ttaagggaactactagacc		To screen for the introduction of the ATP hydrolysis site mutations
B3115	actgcgtgttggtt		To screen for the introductions of the endonuclease site mutations
B3116	tgctgcaagttttgc		To screen for the introduction of the β-clamp binding site mutations
B3117	gacaattaacagaagatgatc		Pairs with B3115 and B3116 for point mutation screening
B3122	accaaacaatccagaggcctt		For *mutL* stretch including either the ATP binding site or the ATP hydrolysis point mutations
B3124	tgacagcctttctacaggcaatag		For *mutL* stretch including either the endonuclease site or the β-clamp binding site mutations
B3128	gtgaatacctgcttttcaaattctt		For analysis of merodiploidy in the β-clamp binding site mutant, pairs with B3110

Introduced mutations are in red. Complementary sequences used to anneal the *mutL* fragments are underlined. Restriction endonuclease sites are indicated in the sequence in italics.

References

Dresser, A.R., Hardy, P.-O., and Chaconas, G. (2009). Investigation of the role of DNA replication, recombination and repair genes in antigenic switching at the *vlsE* locus in *Borrelia burgdorferi*: an essential role for the RuvAB branch migrase. *PLoS Pathogens* 5(12)**,** e1000680.

Purser, J.E., and Norris, S.J. (2000). Correlation between plasmid content and infectivity in *Borrelia burgdorferi*. *Proc Natl Acad Sci U S A* 97(25)**,** 13865-13870.

Taylor, R.G., Walker, D.C., and McInnes, R.R. (1993). *E. coli* host strains significantly affect the quality of small scale plasmid DNA preparations used for sequencing. *Nucleic Acids Res* 21(7)**,** 1677-1678.

Verhey, T.B., Castellanos, M., and Chaconas, G. (2018). Analysis of recombinational switching at the antigenic variation locus of the Lyme spirochete using a novel PacBio sequencing pipeline. *Molecular microbiology* 107(1)**,** 104-115.

Vieira, J., and Messing, J. (1991). New pUC-derived cloning vectors with different selectable markers and DNA replication origins. *Gene* 100**,** 189-194.
