# Supplementary material for: The Putative Endonuclease Activity of MutL Is Required for the Segmental Gene Conversion Events That Drive Antigenic Variation of the Lyme Disease Spirochete
Source: Front Microbiol. 2022 May 19;13:888494. doi: 10.3389/fmicb.2022.888494 (PMC9159922; doi:10.3389/fmicb.2022.888494)
Supplement: Supplementary file 2 [file Data_Sheet_2.PDF]

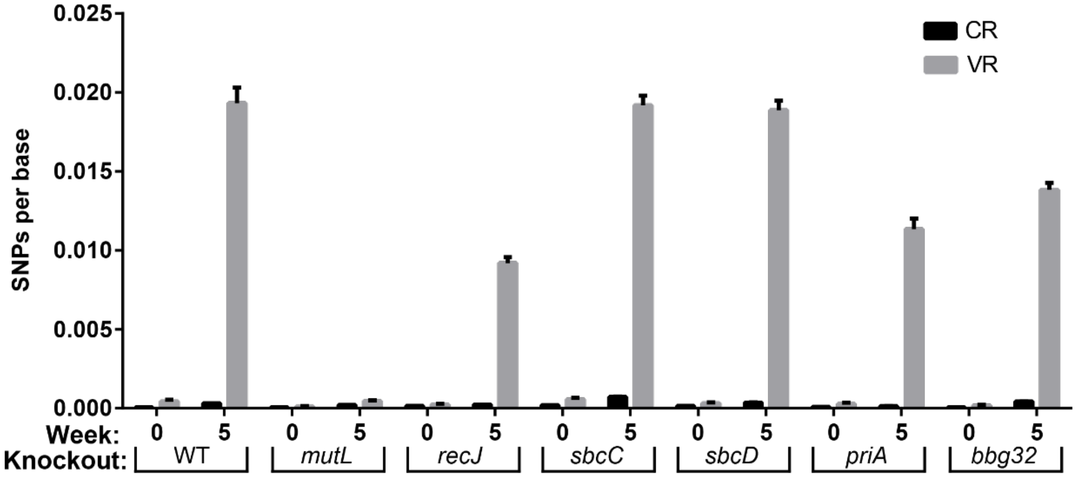

Fig. S1. Frequency of SNPs inside the constant and variable regions of *vlsE*. The frequency of SNPs (including both templated and non-templated) is shown for each gene disruption both pre- and post-infection. The variable region was defined as the smallest region in *vlsE* that contains all of the SNPs possibly templated from the silent cassettes. The constant region is the remainder of the *vlsE* gene in which no templated changes can occur. The mean ± SEM is shown for each bar.

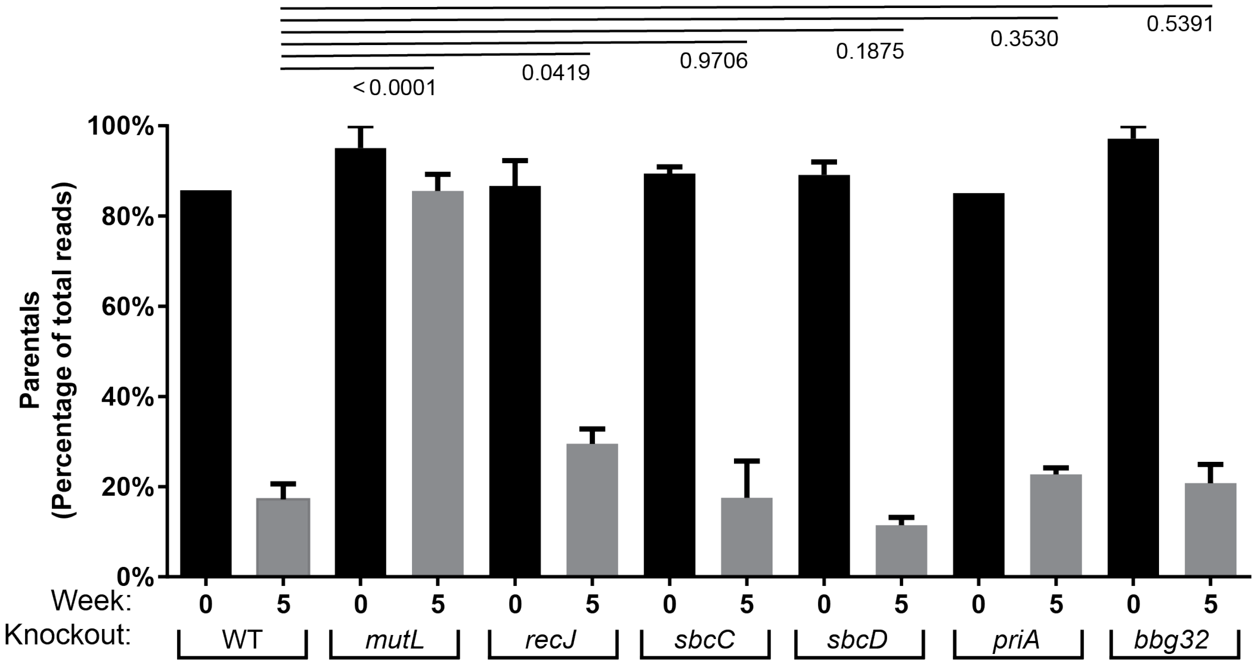

Fig. S2. Frequency of parental *vlsE* sequences. For *vlsE* sequences pre- and post-infection, the fraction of *vlsE* sequences that are identical to the parental *vlsE* sequence is shown, including those that differ by templated sequence changes, non-templated sequence changes, or both. The mean ± SEM is shown for each bar except the zero time for WT and *priA*, where only the value for a single mouse was recovered. *P*-values were determined for each mutant by comparison of the 5-week points with WT using a Bonferroni corrected multiple *t*-test.

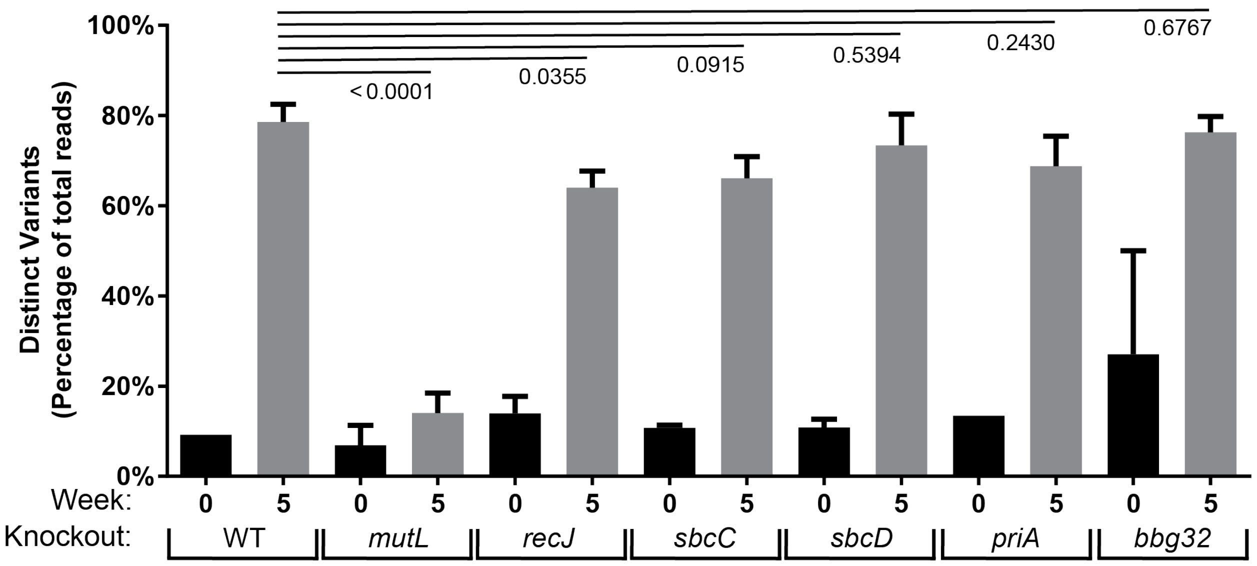

Fig. S3. Distinct variants of *vlsE* sequences. The number of distinct *vlsE* sequences, as a percentage of the total number of *vlsE* sequences is shown for each gene disruption, for both pre- and post-infection samples. 100% denotes that each *vlsE* sequence represents a distinct variant; 0% indicates that there are no *vlsE* sequences other than the parental sequence. The mean ± SEM is shown for each bar except the zero time for WT and *priA*, where only the value for a single mouse was recovered. *P*-values were determined for each mutant by comparison of the 5 week points with WT using a Bonferroni corrected multiple *t*-test.

ATP binding

ATGAACAAAATAAGATTCTTAGATAAATACTTGGTTCAAAAAATAGCAGtAGGAGAATCAATAGACAGGC
1 70

ATP hydrolysis

CATGTTCAATATTAAGGGAACTACTAGACcATTCAATAGATTCTGGAGCTACTAAAATTGAGGTTTTTCTTG
71 142

Endonuclease

GAATTTTTAATCGTTGAAAAAATAAATGAAATTTACTTCATAaACCAACACGCAGTTCACGAAAAAATAATA
1321 1392

β-clamp binding

TATGAAAAACTTAGAAATTCAAAAAAAAATGTTgcAAAACTTgcAGcACCAATTGAATTCACAGTAGTTGAT
1393 1464

Fig. S4. *mutL* point mutations used in this study. ATP binding site corresponding to A17V, ATP hydrolysis site (N34H), endonuclease site (D455N), and β-clamp binding site (Q476A, L479A and V480A). The mutated nucleotides are in red lower-case letters. Underlined are the complementary regions used to assemble the mutated *mutL* genes (see **Table S4** for oligo information). Numbers under the sequences mark the nucleotide position in the *B. burgdorferi* *mutL* gene.

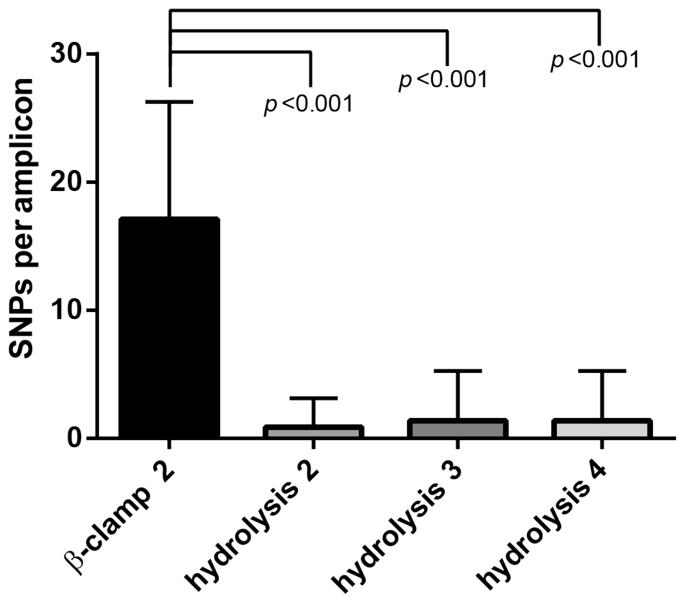

Fig. S5. The effect of mutations in MutL on recombinational switching at *vlsE* as measured by the number of SNPs in the variable region per amplicon, from Sanger sequencing at week 4 in a combined mouse ear and bladder sample. The previously characterized *B. burgdorferi* β-clamp mutant with wild-type *vlsE* switching levels (see **Fig. 8**) was used as a positive control. For the ATP hydrolysis mutants, the previously characterized clone 2 (see **Fig. 8)** as well as two additional mutants (clone 3 and clone 4, see **Table S3**) were used to infect SCID mice as described in Experimental Procedures. Means ± SD are shown. *p* values were determined for individual graphs versus β-clamp binding mutant values using the Kruskal-Wallis test using Dunn's multiple comparisons.
